# miR-138-5p-mediated HOXD11 promotes cell invasion and metastasis by activating the FN1/MMP2/MMP9 pathway and predicts poor prognosis in penile squamous cell carcinoma

**DOI:** 10.1038/s41419-022-05261-2

**Published:** 2022-09-23

**Authors:** Xingliang Tan, Zhenhua Liu, Yanjun Wang, Zhiming Wu, Yuantao Zou, Sihao Luo, Yi Tang, Dong Chen, Gangjun Yuan, Kai Yao

**Affiliations:** 1 Department of Urology, Sun Yat-sen University Cancer Center, Guangzhou, China; 2 State Key Laboratory of Oncology in Southern China, Guangzhou, China; 3 Collaborative Innovation Center of Cancer Medicine, Guangzhou, China; 4 Department of Urology Oncological Surgery, Chongqing University Cancer Hospital, Chongqing, China; 5 Chongqing Key Laboratory of Translational Research for Cancer Metastasis and Individualized Treatment, Chongqing University Cancer Hospital, Chongqing, China

**Keywords:** Penile cancer, Cell invasion

## Abstract

The presence and extent of regional lymph node and distant metastasis are the most fatal prognostic factors in penile squamous cell carcinoma (PSCC). However, the available biomarkers and detailed mechanisms underlying the metastasis of PSCC remain elusive. Here, we explored the expression landscape of HOX genes in twelve paired PSCC tissues, including primary tumors, metastatic lymph nodes and corresponding normal tissues, and highlighted that HOXD11 was indispensable in the progression of PSCC. HOXD11 was upregulated in PSCC cell lines and tumors, especially in metastatic lymph nodes. High HOXD11 expression was associated with aggressive features, such as advanced pN stages, extranodal extension, pelvic lymph node and distant metastasis, and predicted poor survival. Furthermore, tumorigenesis assays demonstrated that knockdown of HOXD11 not only inhibited the capability of cell proliferation, invasion and tumor growth but also reduced the burden of metastatic lymph nodes. Further mechanistic studies indicated that miR-138-5p was a tumor suppressor in PSCC by inhibiting the translation of HOXD11 post-transcriptionally through binding to the 3′ untranslated region. Furthermore, HOXD11 activated the transcription of FN1 to decompose the extracellular matrix and to promote epithelial mesenchymal transition-like phenotype metastasis via FN1/MMP2/MMP9 pathways. Our study revealed that HOXD11 is a promising prognostic biomarker and predicts advanced disease with poor outcomes, which could serve as a potential therapeutic target for PSCC.

## Background

Penile squamous cell carcinoma (PSCC), which accounts for the vast majority of (≥ 95%) penile cancers, is a devastating malignancy in males both physically and psychologically [1–3]. The presence and extent of lymph node metastasis, with a dramatic decline in 5-year overall survival rates from 90% to 29–59%, are the most fatal prognostic factors in PSCC [4–6]. Despite the rapid development of comprehensive therapies [7, 8], the existing treatments are still unsatisfactory and lack available targeted therapies [9]. The detailed mechanisms behind tumor progression remain elusive. Although a recent genetic understanding of PSCC has shown that HRAS mutations, EGFR amplifications, and dysregulation of CAV1 and IDO1 are associated with advanced disease [10–12], most studies lack specific mechanisms owing to the lack of appropriate PSCC cell lines, convincing metastatic experiments in vivo and large-scale clinical validations. Our previous studies have established a molecular stratification to predict high-risk PSCC patients with lymph node metastasis [13, 14], but the mechanism needs to be further explored in depth. Therefore, exploring effective biomarkers in PSCC and clarifying the underlying mechanisms in tumor metastasis to improve outcome are imminent and difficult issues.

Homeobox (HOX) gene clusters encode a series of highly conserved transcription factors that are essential for tumorigenesis by regulating cell differentiation, angiogenesis and metastasis [15, 16]. The dysregulation of HOX genes was associated with numerous solid malignancies and leukemia, suggesting its prominent roles in tumor progression [15–20]. However, the expression pattern of HOX clusters and their oncogenic roles remain unknown in PSCC.

In the present study, we explored the expression landscape of HOX genes in PSCC and highlighted that homeobox D11 (HOXD11) was indispensable in the progression of PSCC. HOXD11, located at chromosome 2q31.1, is involved in neoplastic transformation, especially the processes of tumor invasion and metastasis, in previous studies [16]. Kristina et al. reported that knockdown of the expression of HOXD11 repressed tumor growth and lung metastasis in Ewing’s sarcoma [19]. In addition, the overexpression of HOXD11 was a poor prognostic biomarker in head and neck squamous cell carcinoma and gliomas by promoting tumor proliferation and invasion [17, 20]. Our results first revealed a significant association between HOXD11 expression and clinicopathological features as well as poor outcomes in a large PSCC cohort. We aimed to investigate the biological functions of HOXD11 in PSCC and subsequently explored the potential mechanisms in metastasis.

Herein, we demonstrated that HOXD11 was post-transcriptionally regulated by miR-138-5p at 3′ untranslated region (UTR) and suppressed its translation. Meanwhile, HOXD11 bound to the promoter regions of fibronectin 1 (FN1), activating the expression of MMP2 and MMP9 to decompose the extracellular matrix (ECM) and promoting cell invasion and metastasis through the epithelial mesenchymal transition (EMT) phenotype in vitro and in vivo. These findings provide new insight into HOXD11-mediated PSCC progression and present a potential therapeutic target for tumor metastasis.

## Methods

### Patients, tissue specimens and research ethics

This study was conducted on a total of 267 PSCC patients with well-preserved paraffin-embedded tumor specimens that had been pathologically confirmed according to the TNM Staging System for Penile Cancer (8th ed., 2017) at the Sun Yat-sen University Cancer Center (SYSUCC) from 2003 to 2021. Among them, 12 pairs of matched tissues from pN+ PSCC patients were retrieved for HOX gene expression pattern screening. Then, 93 fresh frozen tumor samples and 21 normal tissues were retrieved from mRNA and protein extraction. The study was approved by the SYSUCC Ethics Committee (GZR2019-167), and informed consent was acquired.

### Real-Time quantitative polymerase chain reaction (qPCR)

The progression target gene screening was based on the mRNA expression panel of the HOX gene cluster. Total RNA extraction, reverse transcription and cDNA amplification were performed according to the standard protocol as described previously. Relative target gene expression was quantified by the 2^(−∆∆Ct)^ method and normalized against GAPDH. Detailed information on primer sequencing is listed in Table S1.

### Western blot (WB)

Tissues and cells were lysed in RIPA buffer (Beyotime) containing 1% protease inhibitors at 4 °C for 30 min, and the concentration of proteins was detected by Comassie Brilliant Blue G250 (Beyotime) according to the manufacturer’s protocol. Equal amounts of protein (30 µg) were separated by 10–15% SDS–PAGE and transferred to PVDF membranes (Pierce Biotechnology). The membranes were blocked with 5% nonfat milk and incubated according to the recommended conditions for the primary and secondary antibodies. The bands were visualized by ECL reagents (EpiZyme). Antibodies and dilutions are listed in Table S2.

### Cell lines and cell culture

The PSCC cell lines Penl1, Penl2, 149rca, 149rm and 156 lm were established in our laboratory as previously reported. The human epidermis keratinocyte cell line (HaCaT) was purchased from the Chinese Academy of Sciences (Shanghai, China). Cell lines were maintained in 10% fetal bovine serum DMEM with 1% penicillin and 1% streptomycin (Gibco), and cells were cultured at 37 °C in a 5% CO_2_ incubator.

### Plasmids, transfection and lentiviral infection

Short hairpin RNA (shRNA) and negative control (NC) sequences were cloned into the lentiviral vector GV248 (hU6-MCS-Ubiquitin-IRES-puromycin) to silence HOX genes (Shanghai Genechem Co., Ltd.). The full-length human HOXD11 gene was amplified and cloned into a vector (pcDNA3.1) to generate HOXD11 overexpression plasmids. Lentiviral packaging and infection were performed using 293 T cells as previously described. To silence FN1, small interfering RNAs (siRNAs) were designed and synthesized by GenePharma (Shanghai, China) and transfected with Lipo8000TM Transfection Reagent (Beyotime). The effective sequences were as follows: shHOXB2, TTACTGAATTAGCGTTTAATC; shHOXD10, TCGTAATGCAGGGTAACTATT; HOXD11-sh1, GGTTTAATGACGTCTCTTCTC; HOXD11-sh2, CGCGAACTGGAACGCGAGTTT; siFN1-F: GCAGCACAACUUCGAAUUATT.

### Cell proliferation and invasion assays

Clone formation assays and Cell Counting Kit-8 (CCK-8) assays were conducted to detect the proliferation potential of PSCC cells in vitro. In brief, 2000 cells were seeded in 6-well plates, and the number of cell colonies was counted after 14 days. Similarly, 1500 cells were cultured with 100 μl medium in 96-well plates and incubated with 10 μl CCK-8 solution (Dojindo, Japan) for 2 h. The absorbance was read on a spectrophotometer at 450 nm for 7 consecutive days. In addition, wound healing assays and Matrigel Transwell invasion assays were used to evaluate the progressive capability of cells as described in our previous study [21].

### Tumor xenograft assays

For the subcutaneous xenograft tumor model, 5–7-week-old BALB/c nude mice (Jiangsu GemPharmatech Co., Ltd.) were randomly divided into two groups (*n* = 6) and were injected subcutaneously with 5 × 10^5^ Penl2-shNC or Penl2-shHOXD11 cells. Tumors were harvested after 3 weeks and subjected to IHC assays. Furthermore, we established a lymph node metastasis xenograft model to explore the spontaneous metastatic capability of PSCC cells. A total of 1.2 × 10^5^ Penl2-shNC or Penl2-shHOXD11 cells (5 mice per group) in 100 μl PBS were injected into the right footpad in nude mice, and popliteal lymph nodes were exposed and harvested after 6 weeks. Tumor volume was calculated as follows: Volume (mm^3^) = 0.5 × length × width^2^. In addition, mice were euthanized when the volume of the tumor exceeded 1500 mm^3^ or weight loss exceeded 15%. The Experimental Animal Ethics Committee of SYSUCC (L102012019002 V) approved the animal experiments.

### Immunofluorescence (IF)

A total of 1000 cells were cultured in 35-mm confocal dishes. After incubation for 24 h, the cells were fixed in 4% paraformaldehyde for 15 min, permeabilized with Triton X-100 (Beyotime) for 10 min and blocked with QuickBlock™ Blocking Buffer (Beyotime) for another 10 min. The cells were then incubated with corresponding primary and secondary antibodies (Table S2) along with DAPI (Sigma, F6057) for visualization using confocal microscopy.

### Immunohistochemistry (IHC) and immunocytochemistry (ICC)

For IHC staining, 4-µm paraffin-embedded tissue sections were processed according to standard pathologic procedures, as previously described [21]. For ICC, cells were seeded on glass slides in 12-well plates and fixed in 4% paraformaldehyde for 15 min. The following steps were similar to the IHC procedures. Pathological diagnosis and IHC staining scores were determined by two independent pathologists (LLL and CKM). If the scores were different, the results were redetermined after discussion. HOXD11 staining scores were multiplied by the staining intensity (0 for faint, 1 for weak staining and 2 for strong staining) and staining area (positive staining proportion of nuclei: 1 for 1–10%, 2 for 11–50%, and 3 for 50% above). The cutoff value was calculated by X-Tile software (version 3.6.1), as previously described [22]. In our cohorts, HOXD11 staining scores of 0–2 points were regarded as low expression, and 3–6 points were regarded as high expression. Besides, the FN1 expressions were reported as negative (no FN1 expression in tumor cells), weak (faint or yellow staining intensity with sporadic expression pattern) and strong (yellow or yellow-brown staining intensity with diffuse expression pattern) on the basis of the staining intensity and pattern.

### miRNA prediction and validation

Two bioinformatics tools, TargetScan [23] and starBase [24] were used to predict the MicroRNAs (miRNAs) for HOXD11. The predicted binding sites of target miRNA and HOXD11 3–UTR are listed in Table S3. MiPure Cell/Tissue miRNA Kit (Vazyme), miRNA 1st Strand cDNA Synthesis Kit (Vazyme) and miRNA Universal SYBR qPCR Master Mix (Vazyme) were used for miRNA extraction and cDNA reverse transcription (primer: 5′ GTCGTATCCAGTGCAGGGTCCGAGGTATTCGCACTGGATACGACCGGCCT 3′) according to the maniscript respectively. The primers of qPCR amplification are listed in Table S4. The expression level and prognostic outcomes of miR-138-5p in pan-cancers were detected by OncomiR [24] and KM Plotter [25] websites. The mimics and inhibitors of miR-138-5p were synthesized by GenePharma Co., Ltd. (Shanghai, China). We also constructed the wild-type and mutant 3′UTR dual luciferase reporter vectors of HOXD11 by RiboBio Co., Ltd. (Guangzhou, China), and the detailed procedures were similarly described in the section of Methods “2.11 Dual Luciferase reporter assay”.

### Dual Luciferase reporter assay

To generate luciferase reporter plasmids, the human FN1 promoter sequences (nucleotide from +1397 to −244) and the human THBS1 promoter sequences (nucleotide from −1040 to +204) were cloned into the pGL3-basic plasmid (Promega). A total of 5 × 10^4^ HOXD11-overexpressing or shHOXD11 cells and corresponding control cells were cultured in 24-well plates in triplicate for 24 h and then cotransfected with pGL3-FN1 or pGL3-THBS1, *Renilla*, and pGL3-basic control plasmids. After transcription for 48 h, luciferase and *Renilla* signals were detected by a Dual Luciferase Reporter Assay Kit (Promega, E1980), and the ratio was calculated and normalized to *Renilla* luciferase activity.

### Chromatin immunoprecipitation-qPCR (ChIP–qPCR)

The JASPAR database was used to predict the binding sites of HOXD11 and the FN1 promoter regions. The UCSC Genome Browser was used to detect the chromatin states of the promoter regions (H3K27Ac, H3KMe1 and H3KMe3). The ChIP assays were performed using the SimpleChIP® Enzymatic Chromatin IP Kit (CST, #9003) according to the manufacturer’s instructions. In brief, HOXD11-Flag overexpression and vector PSCC cells were cross-linked using 1% formaldehyde for 10 min and were lysed and sonocated to fragments of DNA. Samples were purified through immunoprecipitation with Flag antibody (CST, #14793), conjugated with protein A/G beads, and reversal of cross-linking. Eluted DNA fragments were purified and analyzed by qPCR. The primers are listed in Table S5.

### Bioinformatics analysis

RNA sequencing (RNA-seq) was performed to compare the different expression patterns between Penl2 shNC and HOXD11-sh1 cells. R software was used for Gene Ontology (GO) and Kyoto Encyclopedia of Genes and Genomes (KEGG) enrichment analyses using the clusterProfiler package. Gene-set enrichment analysis (GSEA) was conducted by GSEA tools version 4.1 (http://www.broadinstitute.org/gsea), as previously described [21].

### Statistical analysis

Statistical analysis was performed using SPSS software (Version 25.0). Statistics are presented as the means ± SDs. The differences between two groups were analyzed by Student’s t test or one-way ANOVA. The composition ratios were determined by the chi-square test. Survival analysis was performed with Kaplan–Meier survival curves and a multivariate Cox proportional hazards regression model by the forward method. The correlations between genes were analyzed by Pearson correlation analysis. A *p* value < 0.05 was considered significant.

## Results

### HOXD11 is a potential oncogene promoting tumor progression in PSCC

**Fig. 1:**
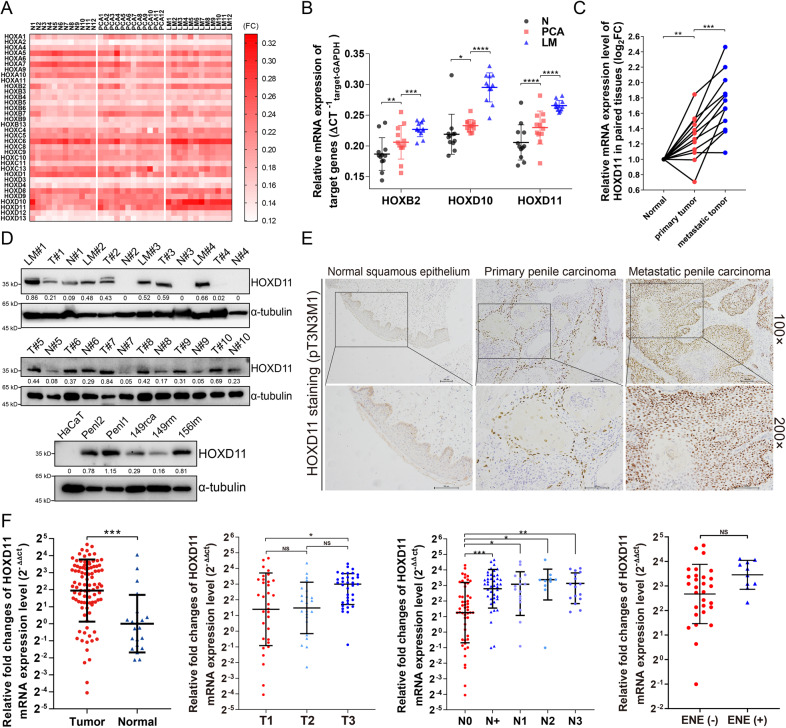
HOX genes expression patterns and clinical significance of HOXD11 in PSCC. Total mRNA was extracted from 12 pN+ PSCC patients with corresponding N, PCA and LM tissues. **A** The HOX genes mRNA expression profile of 12 paired tissues. **B** Among the 34 HOX genes, the mRNA expression of HOXB2, HOXD10 and HOXD11 in tumors especially in LM were striking increased. **C** For each PSCC patients, HOXD11 was overexpressed step by step in PCA and LM than normal tissues. **D** The HOXD11 proteins was increased in tumor tissues and PSCC cell lines. **E** The IHC assays indicated the expression of HOXD11 in paired tissues in a pT3N3M1 PSCC patients. **F** The mRNA levels of HOXD11 were upregulated in 93 tumor tissues compared with those in 21 normal tissues. Overexpression of HOXD11 was associated with poor pT, pN stages and ENE. *p < 0.05; **p < 0.01; ***p < 0.001; ****p < 0.0001. NS not significant, N normal squamous epithelium, PCA primary carcinoma, LM metastatic lymph node, HaCaT human immortalized keratinocytes, IHC immunohistochemistry, ENE extranodal extension, PSCC penile squamous cell carcinoma.

To investigate the oncogenic roles of HOX clusters in PSCC, qPCR was performed to detect the mRNA expression of 34 HOX genes in 12 paired PSCC tissues, including normal squamous epithelium (N), primary penile carcinoma (PCA) and metastatic lymph node tissues (LM). The expression profiles are shown in Fig. 1A. We found a striking increase in the expression of HOXB2, HOXD10 and HOXD11 in tumors, especially with the highest expression in LM tissues, compared with N tissues (Fig. 1B). For each patient, HOXD10 and HOXD11 but not HOXB2 mRNA expression was gradually elevated in LM, PCA and N tissues, indicating the oncogenic potential of tumor aggressiveness in PSCC (Fig. 1C and Fig. S1). Subsequently, we inhibited the expression of three target genes in Penl1 PSCC cells and performed Transwell invasion assays (Fig. S2). We observed a reduction in invasive cells in the HOXD11-silenced group but without significance in the shHOXD10 or shHOXB2 group. These data indicated that HOXD11 might serve as a novel oncogene promoting the progression of PSCC.

### HOXD11 is overexpressed in PSCC tissues and cell lines

To further validate the protein expression of HOXD11 in PSCC, WB assays were performed with tissue samples in pairs from 10 PSCC patients (including 4 pN+ patients) and cell lines. Consistent with the qPCR results, HOXD11 proteins were highly expressed in PSCC cell lines as well as tumors, especially in lymph node metastatic tissues, compared with normal controls (Fig. 1D). In addition, IHC was performed with an mPSCC patient with retroperitoneal lymph node metastases, and we found that HOXD11 was strongly expressed in tumors, especially in metastatic lesions (Fig. 1E). Subsequently, qPCR assays were conducted in a larger cohort of 93 PSCC tumor and 21 normal tissues. The results showed that HOXD11 mRNA levels were overexpressed in tumors (t = 3.312, *p* = 0.001) (Fig. 1F). More importantly, overexpression of HOXD11 was associated with advanced disease, such as T grade, lymph node metastasis and extranodal extension (ENE), indicating the clinical significance of poor outcomes (Fig. 1F).

### Elevated expression of HOXD11 is associated with poor clinical features and survival in PSCC

**Fig. 2:**
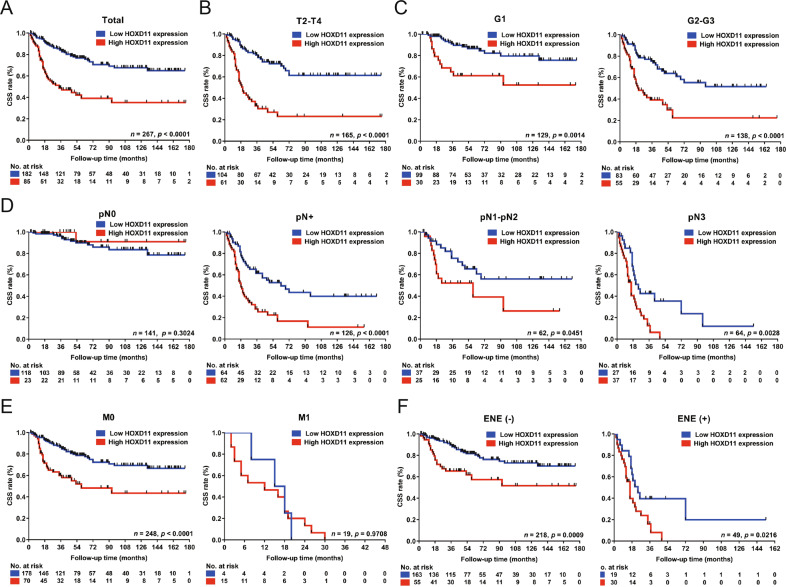
HOXD11 overexpression was associated with poor clinical features and survival in 267 PSCC patients. **A** Kaplan–Meier survival analysis indicated that high HOXD11 expression was associated with lower 5-year CSS rates in the 267 PSCC cohort, **B** pT2-pT4 subgroup, **C** pathological grade subgroup, **D** pN+ subgroup, **E** metastasis subgroup and **F** extranodal extension subgroup of PSCC patients. CSS, cancer-specific survival; PSCC, penile squamous cell carcinoma.

To further determine the association between HOXD11 expression and the clinicopathological features of PSCC, 267 PSCC tumor sections were subjected to IHC staining. The IHC scoring criteria are described in detail in the Methods. HOXD11 was subjected to nuclear staining, and the staining patterns and distribution are shown in Fig. S3. In our cohort, 89/267 (33.3%) patients died of PSCC, with a median follow-up time of 59.1 months. The IHC results indicated that 182 (68.2%) patients had low HOXD11 expression (IHC score 0–2), while 85 (31.8%) patients overexpressed HOXD11 (IHC score 3–6) (Table 1). Chi-square tests demonstrated that high expression of HOXD11 was correlated with poor clinical features, including pT (*p* = 0.003), pN (*p* < 0.001), M status (*p* < 0.001), clinical stage (*p* < 0.001), pathological grade (*p* = 0.011), ENE (*p* < 0.001) and pelvic lymph node metastasis (*p* = 0.038) (Table 1). To further explore the relationship between HOXD11 expression and prognosis, Kaplan-Meier survival analysis was performed and the results revealed that HOXD11 overexpression led to a poor 5-year cancer-specific survival (CSS) rate (*p* < 0.001) (Fig. 2A). Other clinical features, such as pathological grade and pT2-pT4, pN + , M and ENE subgroups, were also associated with a shorter 5-year CSS rate (all *p* < 0.05) (Fig. 2B–F). Moreover, multivariate analysis revealed that HOXD11 expression (*p* = 0.016, HR = 1.759; 95% Cl: 1.112–2.782) and pN status were both independent prognostic indicators of PSCC (Table 2). Taken together, these data suggested that the overexpression of HOXD11 was a novel biomarker contributing to poor clinical prognosis in PSCC.

**Table 1:** Clinicopathological characteristics and the association with HOXD11 expression in 267 PSCC patients.

Variable	PSCC cohort (*N* = 267), %	HOXD11 IHC staining		χ^2^	*p*-value^a^
		Low expression (*N* = 182), %	High expression (*N* = 85), %		
Age				0.019	0.890
<55	143 (53.6)	98 (36.7)	45 (16.9)		
≥55	124 (46.4)	84 (31.5)	40 (15.0)		
pT status				**13.720**	**0.003**^**b**^
≤pT1^c^	102 (38.2)	78 (29.2)	24 (9.0)		
pT2	49 (18.4)	30 (11.2)	19 (7.1)		
pT3	104 (39.0)	71 (26.6)	33 (12.4)		
pT4	12 (4.5)	3 (1.1)	9 (3.4)		
pN status				**38.695**	**0.000**
N0	141 (52.8)	118 (44.2)	23 (8.6)		
N1	32 (12.0)	21 (7.9)	11 (4.1)		
N2	30 (11.2)	16 (6.0)	14 (5.2)		
N3	64 (24.0)	27 (10.1)	37 (13.9)		
Metastasis				**18.650**	**0.000**^**b**^
M0	248 (92.9)	178 (66.7)	70 (26.2)		
M1	19 (7.1)	4 (1.5)	15 (5.6)		
Clinical stage				**33.743**	**0.000**
Stage I	70 (26.2)	58 (21.7)	12 (4.5)		
Stage II	70 (26.2)	57 (21.3)	13 (4.9)		
Stage III	55 (20.6)	36 (13.5)	19 (7.1)		
Stage IV	72 (27.0)	31 (11.6)	41 (15.4)		
Histology				**9.006**	**0.011**
G1	129 (48.3)	99 (37.1)	30 (11.2)		
G2	95 (35.6)	59 (22.1)	36 (13.5)		
G3	43 (16.1)	24 (9.0)	19 (7.1)		
ENE				**23.887**	**0.000**
No	218 (81.6)	163 (61.0)	55 (20.6)		
Yes	49 (18.4)	19 (7.1)	30 (11.2)		
PLNM^d^				**4.298**	**0.038**
No	23 (62.2)	20 (54.1)	3 (8.1)		
Yes	14 (37.8)	7 (18.9)	7 (18.9)		

^a^Chi-square test; ^b^Fisher’s exact test; ^c^Included Ta, Tis and pT1 patients; ^d^A total of 37 PSCC patients underwent pelvic lymph node dissection. *ENE* extranodal extension, *PLNM* Pelvic lymph node metastasis, *PSCC* penile squamous cell carcinoma.

Bold values indicates that the results were statistically significant.

**Table 2:** Univariate and multivariate analyses of HOXD11 expression and clinicopathological features in 267 PSCC patients.

Variable	Total N	Univariate analysis^a^			Multivariate analysis^b^	
		Events (%)	5-year CSS rate (95% Cl)	*p*-value	Hazard ratio (95% Cl)	*p*-value
HOXD11				**0.000**		**0.016**
Low expression	182	43 (23.6)	0.767 (0.698–0.836)		Reference	
High expression	85	46 (54.1)	0.390 (0.267–0.513)		1.759 (1.112–2.782)	
Age				0.400		
<55	143	47 (32.9)	0.679 (0.597–0.761)		Excluded	
≥55	124	42 (33.9)	0.612 (0.512–0.712)			
pT status^c^				**0.000**		0.051
≤pT1	102	19 (18.6)	0.815 (0.729–0.901)	Reference	Reference	–
pT2	49	18 (36.7)	0.547 (0.384–0.710)	**0.002**	1.618 (0.814–3.217)	0.170
pT3	104	41 (39.4)	0.601 (0.497–0.705)	**0.000**	1.815 (1.031–3.197)	**0.039**
pT4	12	11 (91.7)	0.000	**0.000**	3.231 (1.355–7.705)	**0.008**
Histology				**0.000**		0.107
G1	129	27 (20.9)	0.804 (0.730–0.878)	Reference	Reference	–
G2	95	33 (34.7)	0.582 (0.460–0.704)	**0.002**	1.003 (0.577–1.745)	0.990
G3	43	29 (67.4)	0.276 (0.121–0.431)	**0.000**	1.719 (0.915–3.229)	0.092
pN status^d^				**0.000**		**0.000**
N0	141	14 (9.9)	0.903 (0.844–0.962)	Reference	Reference	–
N1	32	12 (37.5)	0.613 (0.431–0.795)	**0.000**	3.728 (1.699–8.176)	**0.001**
N2	30	14 (46.7)	0.503 (0.295–0.711)	**0.000**	4.737 (2.133–10.519)	**0.000**
N3	64	49 (76.6)	0.160 (0.050–0.270)	**0.000**	7.860 (3.073–20.104)	**0.000**
Metastasis				**0.000**		**0.005**
M0	248	70 (28.2)	0.703 (0.638–0.768)		Reference	
M1	19	19 (100)	0.161 (0.028–0.294)		2.557 (1.337–4.890)	
Clinical stage				**0.000**		
Stage I	70	8 (11.4)	0.773 (0.604–0.942)	Reference	Excluded^e^	
Stage II	70	5 (7.1)	0.944 (0.883–1.000)	0.453		
Stage III	55	21 (38.2)	0.614 (0.471–0.757)	**0.000**		
Stage IV	72	55 (76.4)	0.147 (0.045–0.249)	**0.000**		
ENE				**0.000**		0.278
No	218	52 (23.9)	0.755 (0.690–0.820)		Reference	
Yes	49	37 (75.5)	0.161 (0.028–0.294)		1.484 (0.727–3.029)	

^a^Log-rank test; ^b^Cox regression model; ^c^There was no significant difference between pT2 and pT3 subgroup (χ^2^ = 0.001; *p* = 0.979); ^d^There was no significant difference between pN1 and pN2 subgroup (χ^2^ = 0.783; *p* = 0.376); ^e^Clincial stage was excluded from the Cox regression model as it was represented by the TNM stage. *CSS* cancer-specific survival.

Bold values indicates that the results were statistically significant.

### HOXD11 regulates PSCC cell proliferation, migration and invasion in vitro

**Fig. 3:**
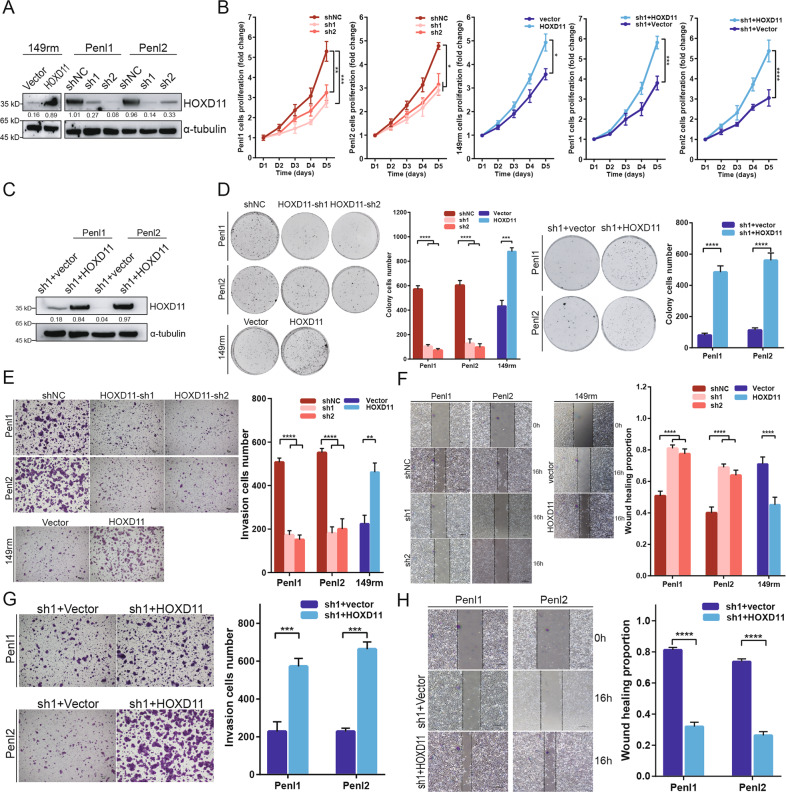
HOXD11 promote cell proliferation and invasion in PSCC in vitro. After overexpressing or silencing HOXD11, PSCC cells were assessed for cell proliferation, clone formation, cell migration and invasion. Besides, rescue experiments were performed to determine to the oncogenic phenotype. **A** WB analysis for HOXD11 expression. **B** CCK-8 assays to determine the proliferative potential of HOXD11. **C** WB was performed to detect the rescue efficacy when HOXD11 was overexpressed in shHOXD11 cells. **D** Colony formation assay, **E** transwell invasion assays **F** and wound healing assays showed that the dysregulation of HOXD11 was involved in tumorigenesis in vitro. **G**, **H** Overexpression of HOXD11 restored the inhibition of cell migration and invasion in shHOXD11 cells. *p < 0.05; **p < 0.01; ***p < 0.001; ****p < 0.0001. Statistics are presented as the means ± SDs of three independent experiments. NS, not significant; NC, negative control; PSCC, penile squamous cell carcinoma.

To explore the oncogenic functions of HOXD11 in PSCC, we first established HOXD11-silenced (Penl1 and Penl2) and HOXD11-overexpressing (149rm) cell lines and validated the protein expression by WB (Fig. 3A). We found that knockdown of HOXD11 in Penl1 and Penl2 cells significantly impaired the proliferation ability (Fig. 3B), reduced the clone and Transwell invaded cell numbers (Fig. 3D, E) and retarded healing of scratch wounds (Fig. 3F) compared with the negative control (NC). Conversely, when we overexpressed HOXD11 in 149rm cells, cell proliferation, migration and invasion were consistently improved (Fig. 3B–F). Moreover, to further validate HOXD11-mediated tumorigenesis in PSCC, we rescued and reupregulated the expression of HOXD11 in Penl1/Penl2-sh1 cells (Fig. 3C). The results indicated that rescuing HOXD11 expression reactivated cell vitality, promoting cell proliferation (Fig. 3B), increasing the number of clones and invaded cells (Fig. 3D, G) and accelerating wound healing (Fig. 3H). These observations revealed that HOXD11 participated in the tumor progression of PSCC.

### Knockdown of HOXD11 inhibits tumor growth and lymph node metastasis in vivo

**Fig. 4:**
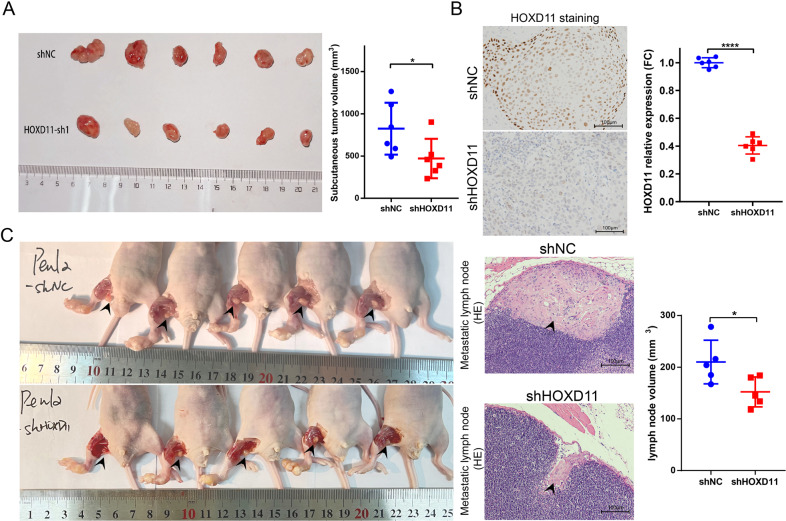
Knockdown of HOXD11 inhibited tumor growth and popliteal lymph node metastasis in vivo. Penl2 HOXD11-sh1 cells were injected subcutaneously to establish xenograft tumor model in BALB/c nude mice (*n* = 6) for three weeks. **A** Knockdown of HOXD11 inhibited tumor growth in vivo. **B** IHC was performed to detect the expression of HOXD11 in two groups. The mRNA expression of HOXD11 was knockdown in subcutaneous tumors of the shHOXD11 group. **C** The effect of HOXD11 on lymph nodes metastasis was explored by an inguinal and popliteal lymph node metastasis model (*n* = 6). Knockdown of HOXD11 inhibited the volume of popliteal lymph node, and HE staining demonstrated the reduction of lymph node metastasis. *p < 0.05. NC, negative control; HE, hematoxylin-eosin; IHC, immunohistochemistry.

To further detect the tumorigenicity ability in vivo, a subcutaneous xenograft tumor model was established in nude mice. After 3 weeks of incubation, the tumors formed by Penl2 HOXD11-silenced cells were significantly smaller than those formed by Penl2-NC cells (Fig. 4A). IHC and qPCR were performed with subcutaneous tumors to identify the knockdown efficiency of HOXD11 (Fig. 4A, B). Then, to better mimic the in vivo metastatic process of PSCC, we established lymph node metastasis models by inoculating shHOXD11 or NC cells into mouse foot pads. We found that the size of popliteal lymph nodes shrank dramatically when HOXD11 expression was knocked down (Fig. 4C). HE staining also confirmed the reduction of lymph nodes with metastases (Fig. 4C). Therefore, our results demonstrated that knockdown of HOXD11 impaired tumor growth and lymph node metastasis in vivo.

### Extracellular matrix regulation contributes to HOXD11-mediated tumor metastasis in PSCC

**Fig. 5:**
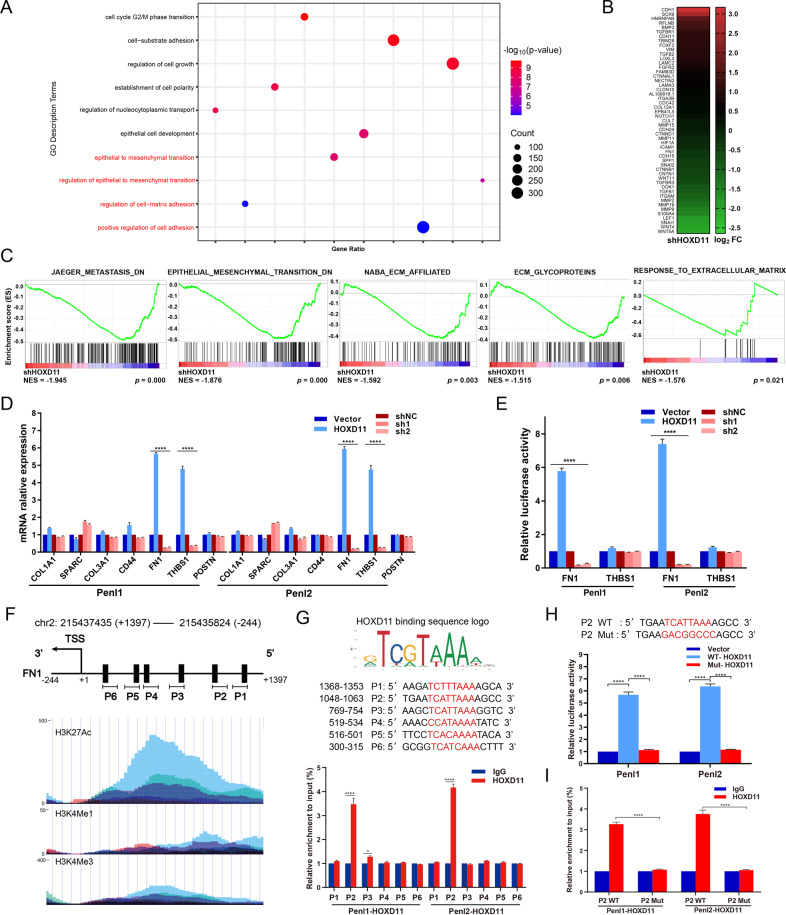
HOXD11 directly bounded with the promoter of FN1 in PSCC. **A** GO enrichment analyses between Penl2 HOXD11-shNC and HOXD11-sh1 cells indicated the significant genes enrichment involving in tumor progression. **B** The differential genes involving in EMT and cell adhesion are shown by heatmap. **C** GSEA analysis showed that knockdown of HOXD11 might promote EMT and tumor metastasis through the degradation of ECM. **D** qPCR analysis of ECM-related markers in the indicated cells. **E** Luciferase reporter assay of FN1 and THBS1 transcriptional activity. **F** Six potential FN1 promoter binding regions with HOXD11 were predicted by JASPAR database. The chromatin stabilization of corresponding regions were detected by H3K27Ac, H3K4Me1 and H3K4Me3 by UCSC Genome Browser. **G** Detail nucleotide binding sequences of FN1 promoter and the CHIP-seq results. **H**, **I** Luciferase reporter and CHIP-seq assays indicated that FN1 transcriptional activity was eliminated when transfection with FN1 promoter with mutant nucleotide binding sequences of P2 sites. *p < 0.05; **p < 0.01; ***p < 0.001; ****p < 0.0001. KEGG, Kyoto Encyclopedia of Genes and Genomes; GO, Gene Ontology; GSEA, gene set enrichment analysis; EMT, epithelial-mesenchymal transition; ECM, extracellular matrix; CHIP, chromatin immunoprecipitation.

To further explore the molecular mechanism by which HOXD11 promotes the progression of PSCC, transcriptome sequencing was performed in shHOXD11-transfected Penl2 and control cells. The GO enrichment and KEGG pathway analyses showed that the biological function of tumor metastasis via epithelial mesenchymal transition (EMT) was significantly enriched when HOXD11 was inhibited in PSCC **(**Fig. 5A and Fig. S4**)**. The heatmap showed that knockdown of HOXD11 in Penl2 cells caused an up-regulation of E-cadherin (encoded by CDH1) with acquisition of an epithelial phenotype and decreased mesenchymal associated proteins such as β-catenin (encoded by CTNNB1) and fibronectin (encoded by FN1) **(**Fig. 5B**)**. Besides, EMT relative transcription factors including Snail1, LEF1 and Snail2 were also inhibited in the shHOXD11 group **(**Fig. 5B**)**. Furthermore, GSEA showed that knockdown of HOXD11 induced the process of EMT through the degradation of extracellular matrix (ECM), which was reported to be a major driver of the loss of adherens junctions in epithelial cells promoting metastasis (Fig. 5C).

To clarify how the transcription factor HOXD11 promoted ECM-mediated tumor progression, qPCR was performed to detect the mRNA expression of downstream ECM-associated targets. The results indicated that overexpression of HOXD11 in Penl1 and Penl2 cells dramatically increased FN1 and THBS1 expression, while FN1 and THBS1 were inhibited correspondingly in HOXD11-silenced cells (Fig. 5D). Dual luciferase reporter assays further demonstrated that HOXD11 overexpression increased whereas HOXD11 knockdown attenuated the transcriptional activation of FN1 in the Penl1 and Penl2 cells (Fig. 5E). However, when the THBS1 promoter plasmid was transfected into HOXD11 overexpression or knockdown cells, the luciferase activity of THBS1 did not change significantly, indicating that HOXD11 did not bind to the THBS1 promoter to regulate its transcription (Fig. 5E). Collectively, these data suggested that HOXD11 might induce FN1 transcription by participating in ECM-mediated EMT and promoting tumor metastasis in PSCC.

### HOXD11 directly binds with the promoter regions of FN1 and upregulates its expression

To further clarify the mechanism underlying the regulation of FN1 expression by HOXD11, we used the JASPAR database to analyze and predict 6 regions (P1-P6) of the FN1 promoter containing HOXD11 binding sites (Fig. 5F, G). The UCSC Genome Browser showed a visible increase in H3K27 acetylation and H3K4 methylation, indicating open and active chromatin in these regions [26], which might include transcription factor-binding sites (Fig. 5F). ChIP-qPCR assays indicated that HOXD11 can directly bind to the promoter of FN1 at P2 sites (Fig. 5G). Then, we reconstituted and cotransfected the FN1 promoter plasmids with mutant P2 binding sites. The results demonstrated that the luciferase activity of the Mut-P2 reporter gene significantly decreased compared with that of the wild-type group (Fig. 5H). Moreover, ChIP–qPCR assays confirmed that HOXD11 did not bind to the mutant P2 regions of the FN1 promoter (Fig. 5I). Taken together, these findings clarified that the HOXD11 transcription factor can specifically bind to FN1 promoter regions at P2 sites and promote its transcription.

### HOXD11 degrade extracellular matrix to promote metastasis via FN1/MMP2/MMP9 axis in PSCC

**Fig. 6:**
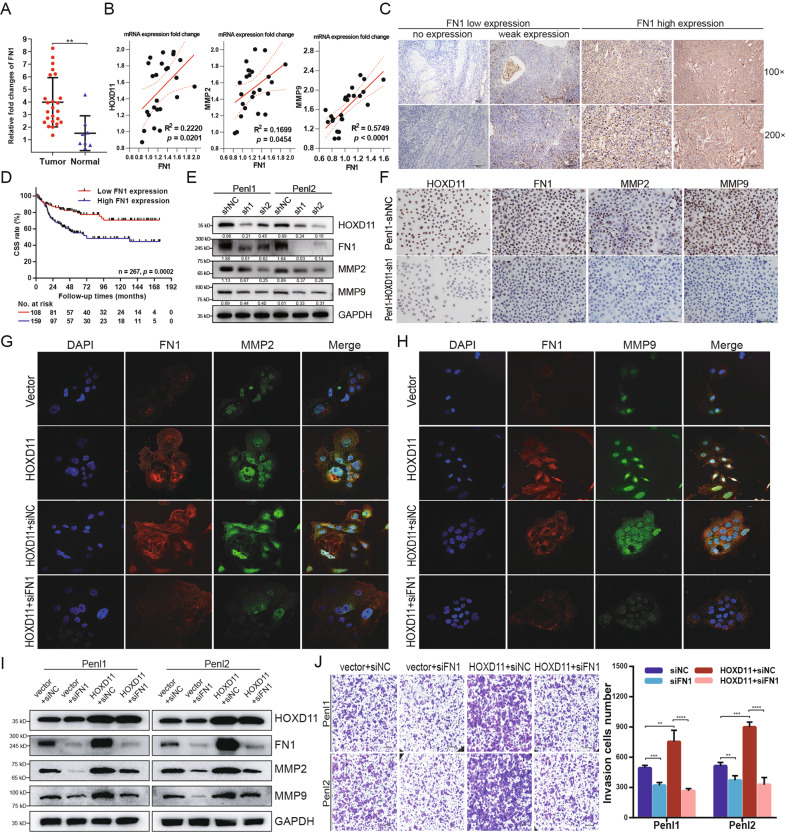
HOXD11 induced the degradation of extracellular matrix via the FN1/MMP2/MMP9 pathways. **A** The mRNA expression of FN1 in 24 PSCC tumors and 8 normal tissues. **B** The expression relationship of target genes in 24 PSCC patients by Pearson correlation analysis. **C** The expression pattern of FN1 in PSCC tumors by IHC assays. **D** High FN1 expression was associated with poor survival in PSCC. **E**, **F** Western bolt and immunocytochemistry showed the decreased protein expression of FN1, MMP2 and MMP9 when knockdown of HOXD11 in PSCC cells. **G**, **H** Immunofluorescence indicated that overexpression of HOXD11 in 149rca cells increased the expression of FN1, MMP2 and MMP9. While the expression of MMP2 and MMP9 were inhibited by siRNA-FN1transfection. **I**, **J** Knockdown of FN1 inhibited cell invasion in PSCC, and undermined the progressive potential of cell invasion in HOXD11 overexpression cells. *p < 0.05; **p < 0.01; ***p < 0.001; ****p < 0.0001. Statistics are presented as the means ± SDs of three independent experiments.

Recent studies have demonstrated that FN1 activates matrix metalloproteinase 2 (MMP2) and MMP9 expression to hydrolyze components of the basement membrane and thus can promote tumor invasion and metastasis in various cancers [27–30]. To explore the expression of FN1 in PSCC and the relationship between HOXD11 and MMPs, qPCR was performed in 24 PSCC patients. We detected that FN1 was upregulated in PSCC tumor tissues (Fig. 6A). Pearson correlation analysis showed that HOXD11 and FN1 (*p* = 0.020; R^2^ = 0.222) as well as FN1 and MMP2 or MMP9 (*p* = 0.045, 0.000; R^2^ = 0.170, 0.575) were positively correlated, indicating linear regulation in PSCC (Fig. 6B). To further demonstrate the clinical significance of FN1 expression in PSCC, IHC was performed in 267 tumor sections (Fig. 6C). The results indicated that FN1 was positively expressed in 243/267 (91.0%) patients and 159/267 (59.6%) of them showed diffuse and strong expression (Fig. S5). Kaplan-Meier survival analysis indicated that PSCC patients with high FN1 expression had poor survival comparing with FN1 low expression (*p* = 0.0002) (Fig. 6D). Subsequently, WB was performed to clarify that knockdown of HOXD11 in Penl1 and Penl2 cells decreased FN1 as well as MMP2 and MMP9 expression (Fig. 6E). Consistent with the WB results, ICC assays also indicated the downregulation of FN1, MMP2 and MMP9 in HOXD11-silenced Penl1 cells (Fig. 6F). In addition, IF assays showed that FN1, MMP2 and MMP9 were increased by overexpression of HOXD11 in 149 BCa cells but were rescued when the expression of FN1 was subsequently suppressed by transfection with si-FN1 (Fig. 6G, H). These data suggested that HOXD11 promoted FN1 transcription and activated the downstream MMP2 and MMP9 proteins in PSCC.

Next, we investigated whether FN1 activation was required for the ability of HOXD11 to promote cell invasion in PSCC. Silencing FN1 not only reduced the number of invasive cells through the Matrigel-coated Transwell chambers in Penl1 and Penl2 cells but also in corresponding HOXD11-overexpressing cells. Moreover, the expression of MMP2 and MMP9 was reversed when FN1 expression was knocked down in HOXD11-overexpressing cells, which suggested the indispensable role of FN1 in HOXD11-mediated tumor progression (Fig. 6I, J). Taken together, we demonstrated that the HOXD11/FN1/MMP2/MMP9 axis was an underlying molecular mechanism promoting tumor invasion and metastasis via an EMT-like phenotype in PSCC.

### miR-138-5p inhibited cell progression by targeting and repressing HOXD11 expression

**Fig. 7:**
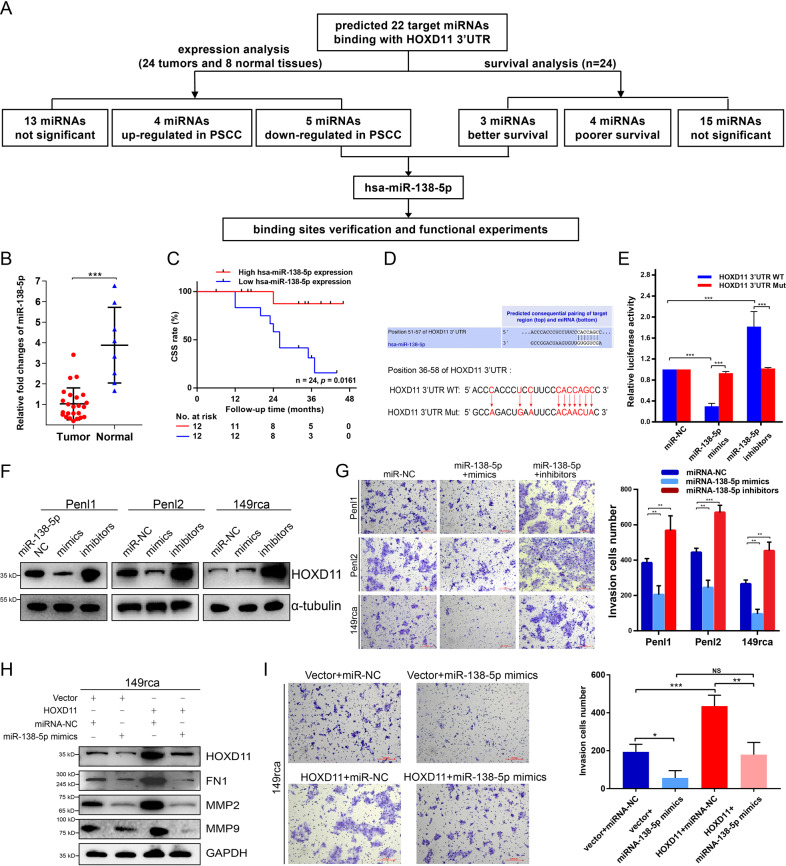
miR-138-5p repressed HOXD11 expression and inhibited cell invasion in PSCC. **A** The flow chart of target miRNA screening. **B**, **C** The expression of miR-138-5p was down-regulated in PSCC tumors and was associated with better survival. **D** TargetScan predicted the bounding sites between miR-138-5p and HOXD11 3′UTR. **E** Luciferase reporter assays indicated that miR-138-5p directly bound with the region of HOXD11 3′UTR. **F**, **G** Cell invasion activity was significantly inhibited in miR-138-5p mimics transfectants, and promoted in miR-138-5p inhibitors transfectants comparing with miR-control transfectant cells. **H**, **I** The invasive cells were reversed when transfecting with miR-138-5p mimics in HOXD11 overexpression 149rca cells.

The role of miRNAs repressing target genes translation post-transcriptionally by targeting the region of the 3′-UTR has been widely reported [31]. To further investigate the upsteam regulator of HOXD11 in PSCC, the miRNAs targeting HOXD11 3′-UTR were predicted by the website tools and the screening process was shown in Fig. 7A. A total of 22 target miRNAs were predicted to bind with HOXD11 3’UTR **(**Table. S3**)**. Then, qPCR was performed in 24 patients to detect the expression of target miRNAs and we found that miR-138-5p was an radical tumor suppressor in PSCC which was down-regulated in tumors and associated with better survival (Fig. 7B, C and Table S5, 6). Besides, miR-138-5p also predicted better prognosis in several cancers in the OncomiR and KM Plotter databases (Fig. S6).

To further verify the regulatory mechanism and functions between miR-138-5p and HOXD11, dual luciferase reporter assays were performed subsequently. Transfecting with miR-138-5p mimics in Penl2 cells significantly decreased luciferase activity of reporter gene with wild-type HOXD11 3′UTR, while increased with the transfection of miR-138-5p inhibitors in comparison with miR-control (Fig. 7D, E). However, this regulatory effect of miR-138-5p was suppressed when the binding site of HOXD11 3’UTR was mutated (Fig. 7D, E), suggesting an exact regulatory relationship between miR-138-5p and HOXD11. WB verified that miR-138-5p mimics inhibited the expression of HOXD11 while by miR-138-5p inhibitors promoted its expression (Fig. 7F). Matrigel-coated Transwell assays showed that the invasive cells decreased or increased when PSCC cells transfected with miR-138-5p mimics or inhibitors respectively (Fig. 7G). More importantly, when miR-138-5p mimics were transfected into HOXD11 overexpression cells, the expression of downstream proteins such as FN1, MMP2 and MMP9 were inhibited (Fig. 7H). The rescue transwell experiments also found that transfection of miR-138-5p mimics reversed the promoted effect of HOXD11 overexpression on cell invasion (Fig. 7I). Taken together, our results demonstrate that miR-138-5p inhibited the translation of HOXD11 post-transcriptionally and regulated the progression of PSCC via FN1/MMP2/MMP9 molecular pathways.

## Discussion

Regional lymph node metastasis, accounting for 20–50% of newly diagnosed patients, is a common and crucial unfavorable prognostic factor in PSCC [1–3]. Even if patients receive contemporaneous radical inguinal lymph node dissection, the 5-year CSS rates (pN1: 74.1%, pN2: 44.7%, pN3: 9.5%) are poor [1, 14]. Moreover, PSCC patients with distant metastasis have ominous findings owing to the low chance of surviving over 5 years [1]. Currently, chemotherapy options and efficacy are limited in PSCC, and there is still a lack of available targeted therapies in clinical practice and relevant basic research [3, 8]. Several studies have reported that RAB20 and IDO1 overexpression promote cell proliferation and induce immunosuppression in PSCC [11, 21]. Other serum biomarkers, such as CXCL5, CXCL13, and CCL20, are associated with nodal metastasis in small cohorts [32–34]. The mechanisms underlying tumor metastasis are uncertain and have become an imminent issue.

HOX genes cluster encode a highly conserved family of transcription factors which have widely reported in neoplastic transformation especially the processes of tumor invasion and metastasis [15, 16]. To identify the role of HOX genes cluster in the progression of PSCC, we performed qPCR with a panel of 34 HOX genes in 12 paired PSCC patients, including normal, primary tumor and metastatic lymph node tissues, and determined that HOXD11 was upregulated and involved in tumor metastasis. Further qPCR, WB and IHC assays confirmed that HOXD11 was overexpressed in PSCC cell lines and tumor samples, especially with the highest expression in corresponding metastatic lymph node tissues. Our results first suggested that the HOXD11 transcription factor, as a potential oncogene, was upregulated in PSCC, especially in metastatic lymph nodes, suggesting a dominant role in the process of PSCC metastasis.

As previously described, HOXD11 is involved in tumorigenesis and tumor progression in several cancers. Harada and Xu et al. found that hypermethylation of HOXD11 in the oral epithelium was an early dangerous event in lung cancer [35, 36]. Similarly, high methylation levels of HOXD11 are also regarded as poor indicators in breast cancer and ovarian cancer [37, 38]. In the regulation of transcription, HOXD11 overexpression participated in head and neck squamous cell carcinoma, laryngeal squamous cell carcinoma, glioma and hemangioblastoma by promoting cell proliferation, cell migration and angiogenesis [15, 17, 20, 39]. In addition, Heyking et al. demonstrated that HOXD11 promoted lung metastasis in Ewing sarcoma [19]. However, the clinical significance and biological functions of HOXD11 in PSCC have not been elucidated.

To this aim, we performed IHC to detect HOXD11 expression in a large PSCC cohort. We detected that HOXD11 overexpression served as an independent prognostic biomarker predicting shorter survival. More importantly, high HOXD11 expression was positively correlated with aggressive metastatic features such as advanced pN stages, extranodal extension, pelvic lymph node and distant metastasis. Our large-scale results first supported that HOXD11 was an aggressive clinical indicator in PSCC. Subsequently, the biological functions of HOXD11 were explored in our newly established PSCC cell lines. Similar to previous findings, HOXD11 knockdown or overexpression inhibited or promoted colony formation, cell proliferation, migration and invasion in vitro and subcutaneous tumor growth in vivo. Uniquely, we employed the footpad xenograft model to simulate the metastatic pattern of PSCC for the first time. The observations further demonstrated that HOXD11 not only inhibited tumorigenicity but also reduced regional lymph node metastatic burden in vivo. Consequently, we clarified the functional role of HOXD11 in tumor progression and metastasis and explored the underlying molecular mechanisms.

To understand the downsteam mechanisms of HOXD11, we first analyzed the differentially expressed mRNA-seq between HOXD11-silenced Penl2 and negative control cells by functional enrichment analysis and found that EMT was involved in the tumor progression of PSCC. EMT is an evolutionarily conserved process in tumor metastasis that enhances mobility, invasion, and resistance to apoptotic stimuli [40]. EMT-derived tumor cells acquire stem cell properties by reducing the polarity of epithelial cells and inducing mesenchymal, fibroblast-like properties [41]. One of the hallmark features of EMT is the degradation of ECM to decrease adherent junctions losing epithelial integrity, which was reminded by the following GSEA (Fig. 5C).

Subsequently, we explored the specific downstream targets by which the transcription factor HOXD11 mediated ECM degradation by qPCR, dual luciferase and ChIP-qPCR assays. Previous studies have indicated that several cell adhesion molecules and ECM components, including collagen 1 and 3 (encoded by COL1A1 and COL3A1), fibronectin (encoded by FN1), periostin (encoded by POSTN) and ECM-associated regulatory proteins (SPARC, THBS1 and CD44), modulate EMT-like transformation and can be regulated by transcription factors such as Twist, Slug and Snail [42, 43]. Our results demonstrated that HOXD11 directly bound to FN1 promoter regions and promoted the expression of FN1, which is a vital component of ECM in shaping the tumor microenvironment to promote metastasis.

FN1 is an ECM glycoprotein participating in cell proliferation, oncogenic transformation and EMT [44]. FN1 is overexpressed in multiple cancer types and is associated with tumor metastasis [27–30, 44–47]. For instance, FN1 increased the expression of VEGF to promote EMT and lymph node metastasis through FAK activation in oral squamous cell carcinoma [27, 47]. In addition, FN1 is involved in the maintenance of the FN1 receptor integrin β1, inducing immunosuppression and promoting progression in glioma [48]. In this study, we first demonstrated that silencing FN1 inhibited the capability of cell invasion in PSCC by Transwell assays. More importantly, knockdown of FN1 also relieved the aggressiveness in HOXD11-overexpressing cells, indicating that FN1 was an essential and indispensable mediator in HOXD11-mediated PSCC tumor progression.

Next, we investigated the detailed mechanisms of HOXD11/FN1-induced ECM degradation in PSCC. Numerous studies have clarified that one of the drivers of EMT-associated ECM degradation is proteolytic digestion by matrix metalloproteinases (MMPs) [29, 44]. FN1 upregulation has been reported to activate MMP2 and MMP9 via the FAK and PI3K/Akt pathways, promoting tumor metastasis in lung, gastric, breast, ovarian and cervical cancer [49–51]. In addition, fibronectin proteins, similar to extracellular glues, can specifically bind with a large number of molecules, such as other components of the extracellular matrix, cell adhesion molecules and MMPs, to modulate the progressive tumor microenvironment [44]. Therefore, we detected the expression levels of HOXD11, FN1, MMP2 and MMP9 in PSCC patients and verified that overexpression of FN1 was positively correlated with MMP2 and MMP9. WB and IF assays indicated that HOXD11 promoted FN1 transcription and induced the expression of MMP2 and MMP9 to degrade ECM and improve metastasis via an EMT-like phenotype in PSCC.

Finally, we also searched for the potential upstream regulator targeting to HOXD11. MiRNAs are dominate small noncoding RNAs that regulate epigenetic processes via interference of transcription and translation to silence gene expression [31]. However, miRNA-mediated the regulation of HOXD11 has not been reported yet. In this study, we found that miR-138-5p decreased in normal tissues comparing with PSCC tumors. Besides, the up-regulated of miR-138-5p was associated with better prognosis in PSCC, which was consistent in bladder cancer, cervical squamous cell carcinoma and hepatocellular carcinoma (Fig. S6). More importantly, miR-138-5p bound to the 3’UTR of HOXD11 and repressed HOXD11 translation post-transcriptionally. MiR-138-5p served as a tumor suppressor inhibiting the HOXD11-mediated progression of PSCC.

In summary, we explored the available oncogene HOXD11, which is overexpressed in PSCC and is associated with lymph node metastasis and poor patient prognoses. Low expression of miR-138-5p in tumors leads to overexpression of HOXD11, which induced FN1 transcription and increased MMP2 and MMP9 expression to degrade ECM and promoted tumor metastasis in vivo and in vitro. We demonstrated that HOXD11 is a novel therapeutic target mediated by miR-138-5p promoting tumor metastasis of PSCC via the FN1/MMP2/MMP9 molecular pathway.

## Conclusions

In this study, we explored the available oncogene HOXD11, which is overexpressed in PSCC and is associated with lymph node metastasis and poor patient prognoses. HOXD11 was mediated by miR-138-5p and induced FN1 transcription and increased MMP2 and MMP9 expression to degrade ECM and promoted tumor metastasis in vivo and in vitro. Our studies provide valuable insights into the molecular mechanism of tumor metastasis and suggest a therapeutic target in PSCC.

## Supplementary information

Reproducibility checklist

Supplementary Materials

## Data Availability

The original western blots of the article are shown in Fig. S7.The datasets supporting the conclusions of this article are available from the corresponding author on reasonable request. The authenticity of this article has been validated by uploading the key raw data onto the Research Data Deposit platform RDDB2022534096 (www.researchdata.org.cn).
