# Supplementary material for: miR-138-5p-mediated HOXD11 promotes cell invasion and metastasis by activating the FN1/MMP2/MMP9 pathway and predicts poor prognosis in penile squamous cell carcinoma
Source: Cell Death Dis. 2022 Sep 23;13(9):816. doi: 10.1038/s41419-022-05261-2 (PMC9508180; doi:10.1038/s41419-022-05261-2)
Supplement: Supplementary file 2 — Supplementary Materials [file 41419_2022_5261_MOESM2_ESM.docx]

**miR-138-5p-mediated HOXD11 promotes cell invasion and metastasis by activating the FN1/MMP2/MMP9 pathway and predicts poor prognosis in penile squamous cell carcinoma**

**Supplementary Materials**

**
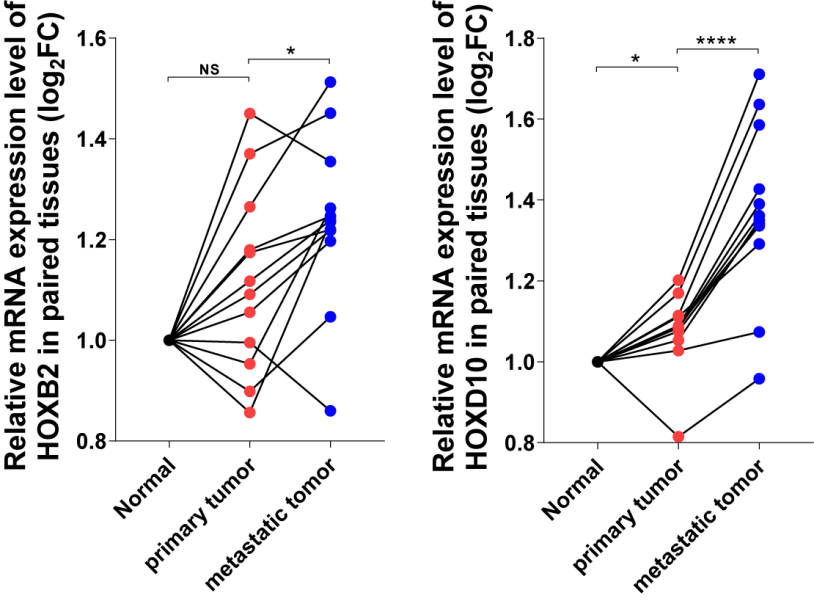
**

***Fig. S1 The mRNA expression of HOXB2 and HOXD10 in the 12 pN+ PSCC patients.***

The trend of HOXB2 and HOXD10 mRNA expression of each pN+ PSCC patients. NS, not significant, PSCC, penile squamous cell carcinoma.


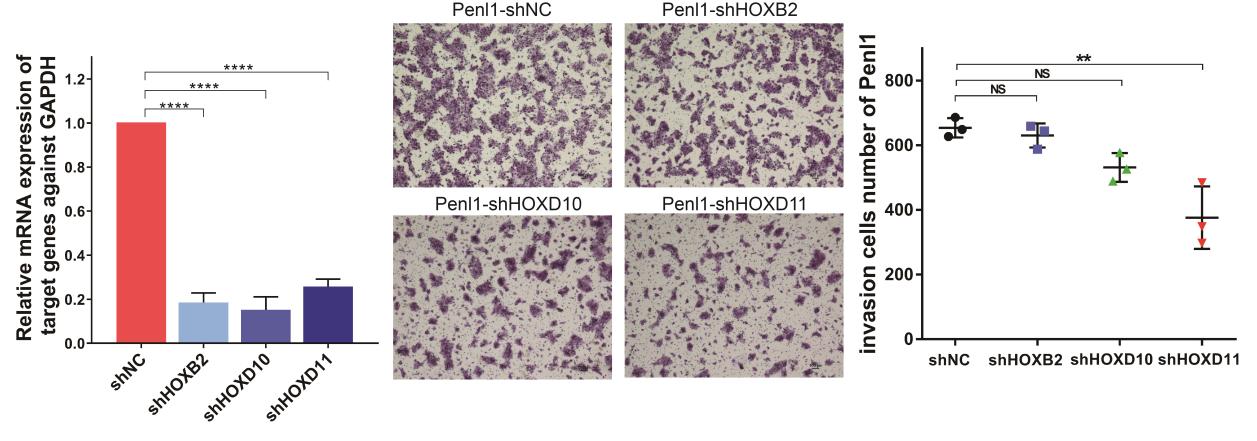


***Fig. S2 Invasive potential of HOX genes in PSCC.***

qPCR indicated the knockdown efficency of target genes. (B, C) Transwell assays indicated that knockdown of HOXD11 in Penl1 cells inhibited cell invasion significantly comparing with shHOXB2 and shHOXD10 groups. total of 259 PSCC patients were divided into the high and low RAB20 expression groups. NC, negative control; NS, not significant, PSCC, penile squamous cell carcinoma.

***
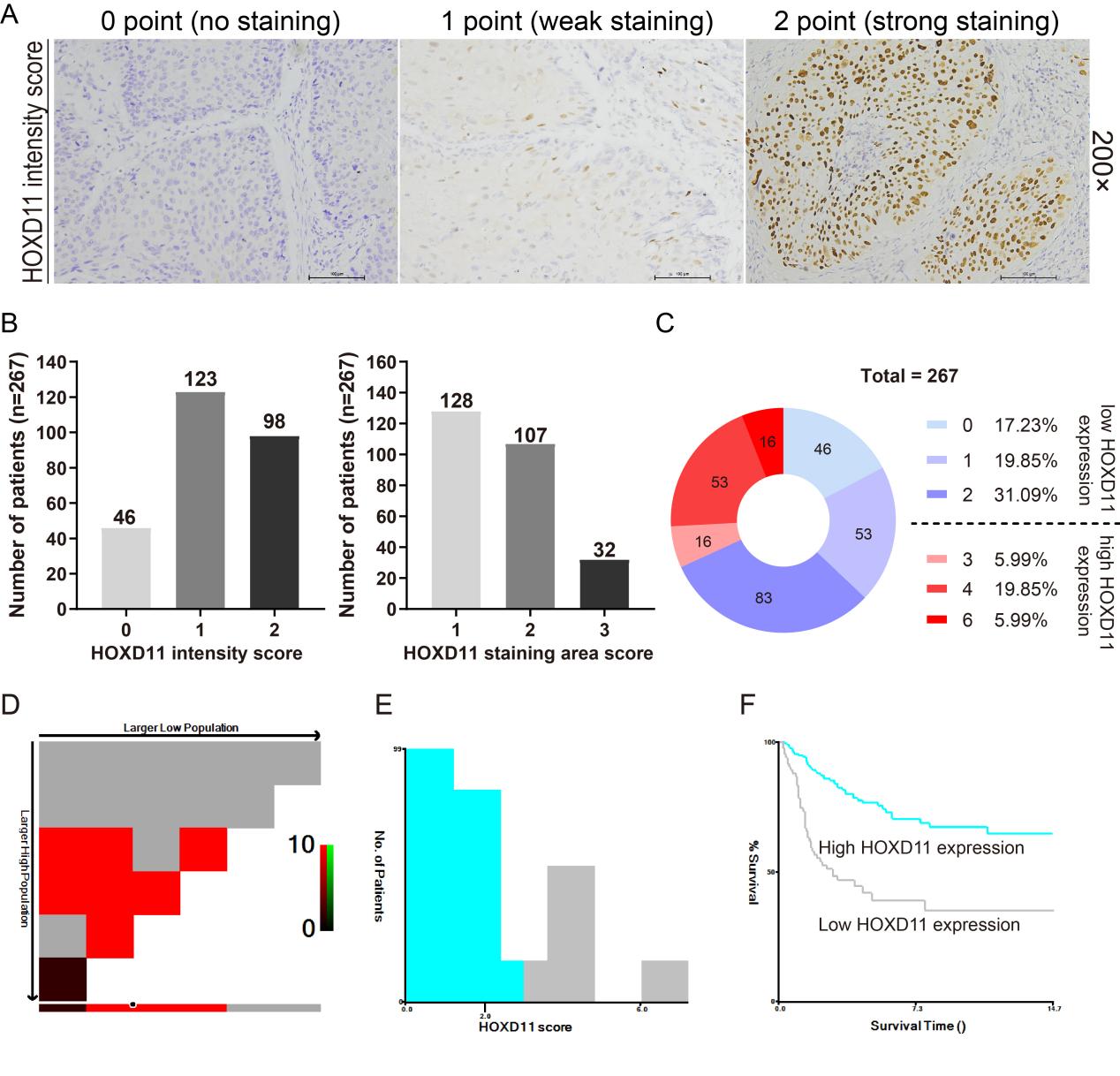
***

***Fig. S3 The distribution of IHC scores and cutoff value of HOXD11 in the PSCC cohort.***

(A) The expression pattern of HOXD11 protein in tumor by IHC. The IHC score of HOXD11 were multiplied by the staining intensity (0 for no staining, 1 for weak staining, 2 for strong staining) and the staining area (1 for 1-10%, 2 for 11-50%, and 3 for 50% above). (B) The frequency of HOXD11 intensity scores and staining scorse are shown. (C) The pie graph showed the distribution of HOXD11 IHC scores in the PSCC cohort. (D) The diagram displayed the χ^2^ log-rank values when the patients were divided into two groups. The optimal cutoff value (H score= 2, 0-2 points were low HOXD11 expression and 3-6 points were high HOXD11 expression) is highlighted by the black spot on the lower axis, (E) and the distribution is displayed in a histogram of the cohort by the X-tile software. (G) Kaplan-Meier survival analysis indicated that high HOXD11 expression is associated with poor survival.


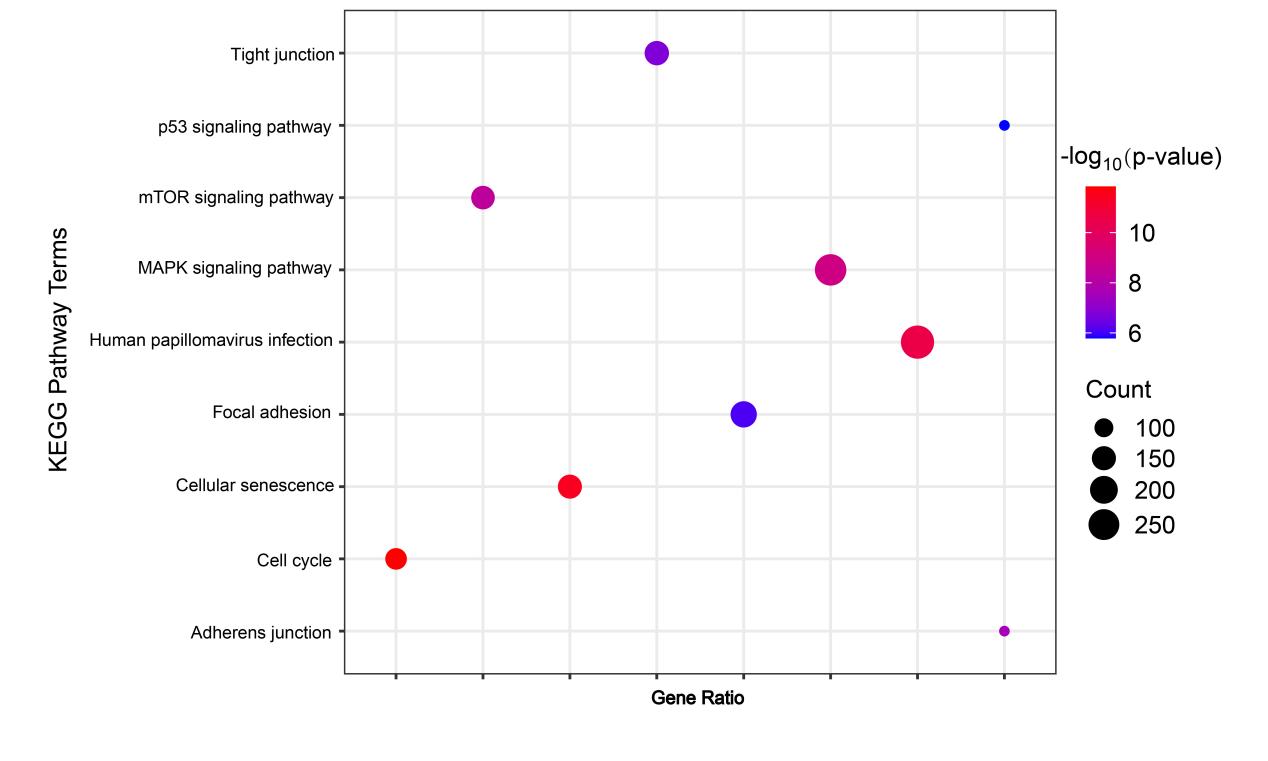


***Fig. S4 The KEGG enrichment analyses between HOXD11-silenced and Penl2 control cells.***

***
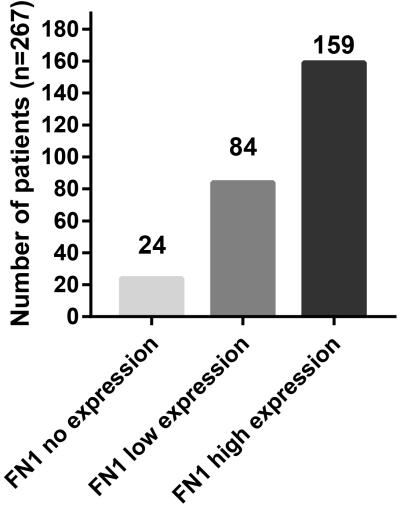
***

***Fig. S5 The distribution of FN1 expression in the PSCC cohort.***

***
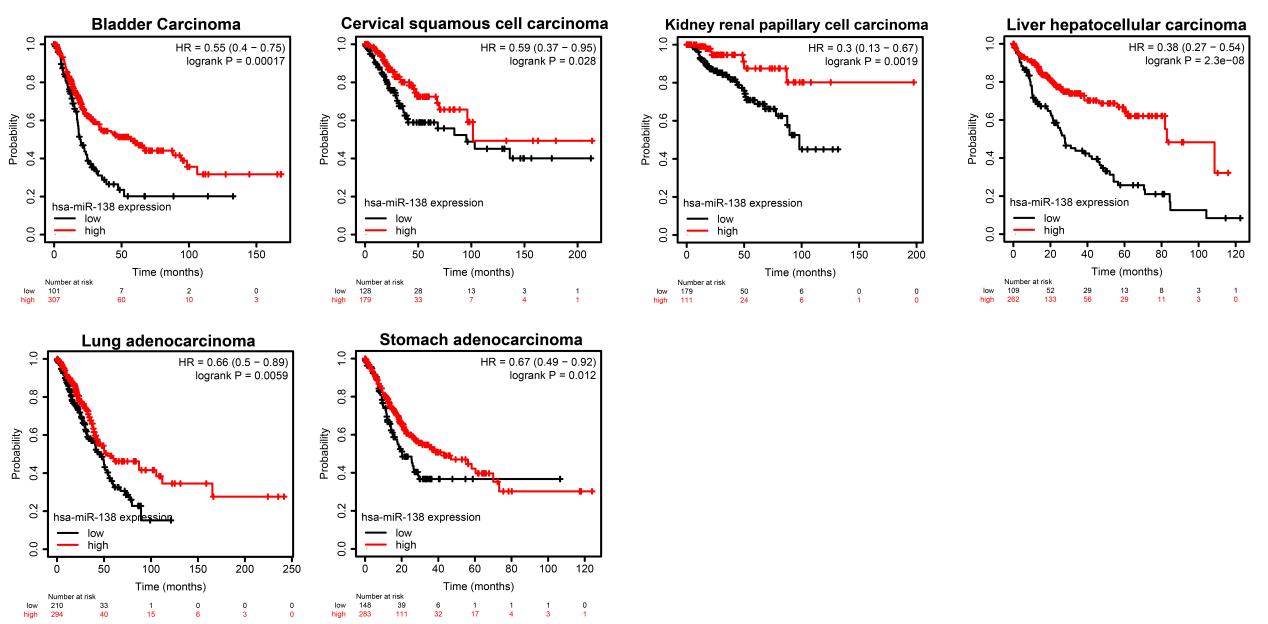
***

***Fig. S6 miR-138-5p overexpression was associated with better clinical outcomes in several cancers.***

Kaplan–Meier survival analysis was performed to detect the survival in Pan-cancers and the expression of miR-138-5p from the KM Plotter database. High miR-138-5p expression was significantly associated with better overall survival in bladder cancer, cervical squamous cell carcinoma, kidney papillary cell carcinoma, hepatocellular carcinoma, lung adenocarcinoma and stomach adenocarcinoma.

***
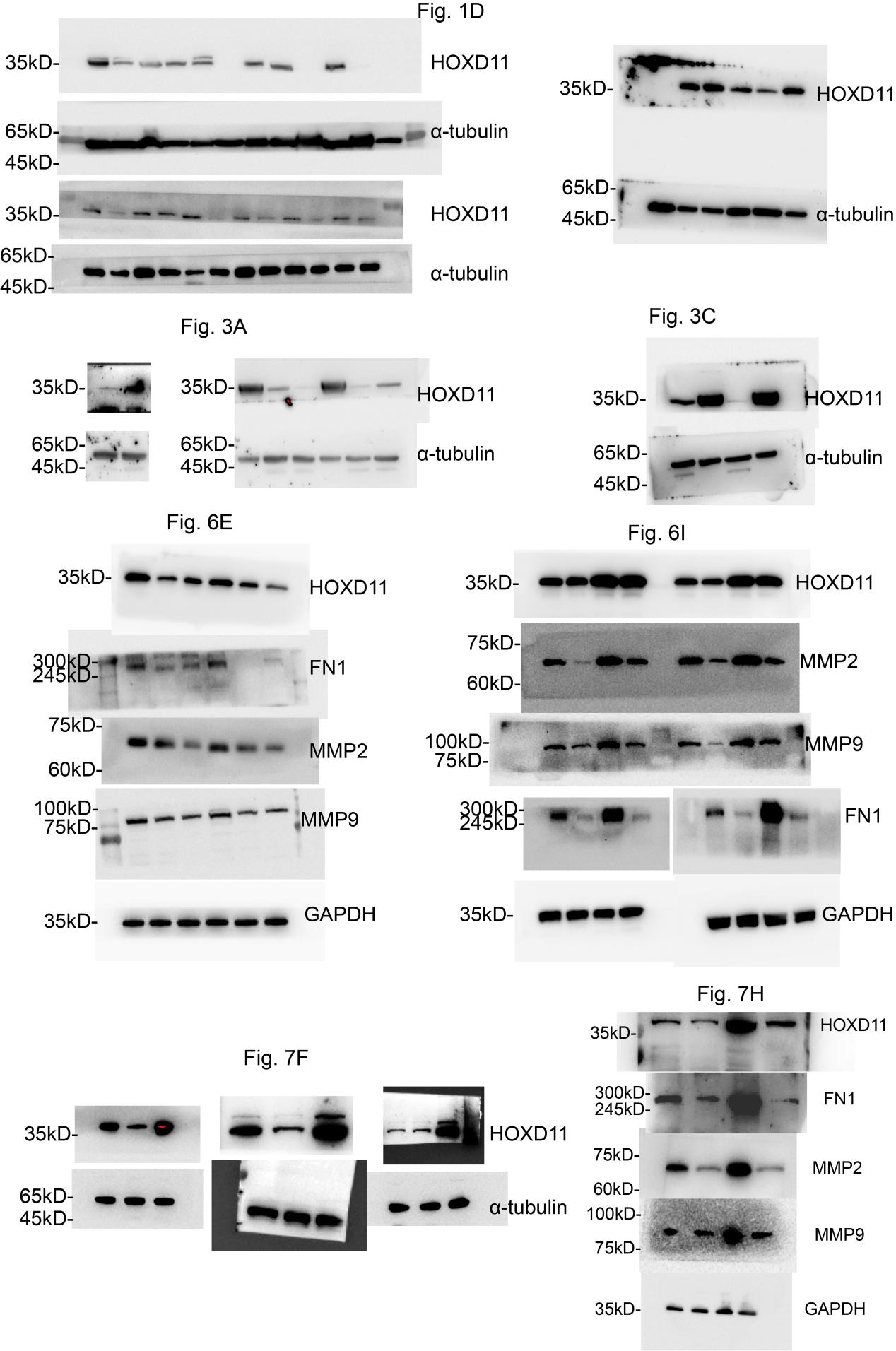
***

***Fig. S7 The original western blots of the article.***

**Table S1.** The primer sequences of HOX genes for qPCR assay.

| **Primer name** | **Forward sequence (5' -> 3')** | **Reverse sequence (5' -> 3')** |
| --- | --- | --- |
| HOXA1 | TCCTGGAATACCCCATACTTAGC | GCACGACTGGAAAGTTGTAATCC |
| HOXA2 | CCCCTGTCGCTGATACATTTC | TGGTCTGCTCAAAAGGAGGAG |
| HOXA4 | ATAACGGAGGGGAGCCTAAG | GCTCAGACAAACAGAGCGTG |
| HOXA5 | AACTCATTTTGCGGTCGCTAT | TCCCTGAATTGCTCGCTCAC |
| HOXA6 | TCCCGGACAAGACGTACAC | CGCCACTGAGGTCCTTATCA |
| HOXA7 | TCGTATTATGTGAACGCGCTT | CAAGAAGTCGGCTCGGCATT |
| HOXA9 | TACGTGGACTCGTTCCTGCT | CGTCGCCTTGGACTGGAAG |
| HOXA10 | CTCGCCCATAGACCTGTGG | GTTCTGCGCGAAAGAGCAC |
| HOXA11 | CCCGCAGTCTCGTCCAATTT | AGGCTGTCTCGAAAAACTGGT |
| HOXB2 | CGCCAGGATTCACCTTTCCTT | CCCTGTAGGCTAGGGGAGAG |
| HOXB3 | AACGCCTTACACTCCATGACC | GACGTGCGGCTCATACTCG |
| HOXB4 | CGTGAGCACGGTAAACCCC | CGAGCGGATCTTGGTGTTG |
| HOXB5 | AGCTCCAGCGCCAATTTCA | TCCGGCCCGGTCATATCAT |
| HOXB6 | GTGCTCCACTCCGGTCTAC | GTAACGTGTGTATGTCTGGCG |
| HOXB7 | CGAGTTCCTTCAACATGCACT | TTTGCGGTCAGTTCCTGAGC |
| HOXB9 | CCGTCTACCACCCTTACATCC | CGTAGCCGGGTCTTTGATTAG |
| HOXB13 | CCAGTTACCTGGACGTGTCTG | GGACCTGGTGGGTTCTGTTC |
| HOXC4 | GCCAGCAAGCAACCCATAGT | CCTTCTCCTTCGGGTCAGGT |
| HOXC5 | AGAGCCCCAATATCCCTGC | CGGTGGGAAAGTGATGCTT |
| HOXC6 | ACAGACCTCAATCGCTCAGGA | AGGGGTAAATCTGGATACTGGC |
| HOXC8 | ACCGGCCTATTACGACTGC | TGCTGGTAGCCTGAGTTGGA |
| HOXC9 | ACTCGCTCATCTCTCACGACA | GACGGAAAATCGCTACAGTCC |
| HOXC10 | ACATGCCCTCGCAATGTAACT | GAGAGGTAGGACGGATAGGTG |
| HOXC11 | AGTTGCACTTACTACATGCCC | GGCCGAGTAGGGATAGGAGA |
| HOXC13 | GCCGTCTATACGGACATCCC | GGTAGGCGCAAGGCTTCTG |
| HOXD1 | CGGGTCTCACGTCCACTAC | GATGCGGTCTGGAAAGCAC |
| HOXD3 | CAAGGGCATCCTGCACTCG | TATTGGGCTGTGATTTGGCGA |
| HOXD4 | CCCTCCGTGCGAGGAGTAT | GAAAGGCTGCTCACCGAAGT |
| HOXD8 | AGAAGAATCGAGGTTTCCCACG | TCCTTTTTCGTTTCCCCGTCC |
| HOXD9 | GGACTCGCTTATAGGCCATGA | GCAAAACTACACGAGGCGAA |
| HOXD10 | AGCGCAACAAACTCATTTCGG | GGGTTCTCAACGGGACAAGAC |
| HOXD11 | TCGACCAGTTCTACGAGGCA | AAAAACTCGCGTTCCAGTTCG |
| HOXD12 | CTCTCAAAGCGGCCAAGTATG | CTGCTTCGTGTAGGGTTTCC |
| HOXD13 | CTTCGGCAACGGCTACTACAG | TGACACGTCCATGTACTTCTCC |

**Table S2.** The information and application of antibodies.

| Gene | Company | Catalog number | Application | Dilution |
| --- | --- | --- | --- | --- |
| HOXD11 | Signalway Antibody | #36925 | WB | 1:1000 |
| HOXD11 | Proteintech | 18734-1-AP | IHC | 1:200 |
| α-tubulin | CST | #2144 | WB | 1:1000 |
| FN1 | Proteintech | 15613-1-AP | WB | 1:1000 |
| FN1 | Proteintech | 15613-1-AP | IF | 1:200 |
| FN1 | Proteintech | 66042-1-Ig | IF | 1:200 |
| FN1 | Proteintech | 66042-1-Ig | IHC | 1:600 |
| MMP2 | Proteintech | 10373-2-AP | WB | 1:1000 |
| MMP2 | Proteintech | 10373-2-AP | IF | 1:200 |
| MMP2 | Proteintech | 10373-2-AP | IHC | 1:400 |
| MMP9 | Proteintech | 10375-2-AP | WB | 1:1000 |
| MMP9 | Proteintech | 10375-2-AP | IF | 1:200 |
| MMP9 | Proteintech | 10375-2-AP | IHC | 1:1000 |

**Table S3.** The predicted consequential pairing of HOXD11 3’UTR and target miRNAs.

| **Target miRNA** | **Target miRNA sequences (5' -> 3')** | **Position of binding sequences of HOXD11 3’UTR (5' -> 3')** |
| --- | --- | --- |
| hsa-miR-138-5p | A**GCUGGU**GUUGUGAAUCAGGCCG | Position 51-57 of HOXD11 3' UTR  ACCCACCCUCCUUCC**CACCAGC**C |
| hsa-miR-20b-5p | C**AAAGUGC**UCAUAGUGCAGGUAG | Position 1109-1116 of HOXD11 3'UTR  GGUGAGGGCUCUACU**GCACUUUA** |
| [hsa-miR-93-5p](http://www.mirbase.org/cgi-bin/mirna_entry.pl?acc=hsa-miR-93-5p) | C**AAAGUGC**UGUUCGUGCAGGUAG | Position 1109-1116 of HOXD11 3'UTR  GGUGAGGGCUCUACU**GCACUUUA** |
| [hsa-miR-519d-3p](http://www.mirbase.org/cgi-bin/mirna_entry.pl?acc=hsa-miR-519d-3p) | C**AAAGUGC**CUCCCUUUAGAGUG | Position 1109-1116 of HOXD11 3'UTR  GGUGAGGGCUCUACU**GCACUUUA** |
| [hsa-miR-17-5p](http://www.mirbase.org/cgi-bin/mirna_entry.pl?acc=hsa-miR-17-5p) | C**AAAGUGC**UUACAGUGCAGGUAG | Position 1109-1116 of HOXD11 3'UTR  GGUGAGGGCUCUACU**GCACUUUA** |
| [hsa-miR-106a-5p](http://www.mirbase.org/cgi-bin/mirna_entry.pl?acc=hsa-miR-106a-5p) | A**AAAGUGC**UUACAGUGCAGGUAG | Position 1109-1116 of HOXD11 3'UTR  GGUGAGGGCUCUACU**GCACUUUA** |
| [hsa-miR-20a-5p](http://www.mirbase.org/cgi-bin/mirna_entry.pl?acc=hsa-miR-20a-5p) | U**AAAGUGC**UUAUAGUGCAGGUAG | Position 1109-1116 of HOXD11 3'UTR  GGUGAGGGCUCUACU**GCACUUUA** |
| [hsa-miR-106b-5p](http://www.mirbase.org/cgi-bin/mirna_entry.pl?acc=hsa-miR-106b-5p) | U**AAAGUGC**UGACAGUGCAGAU | Position 1109-1116 of HOXD11 3'UTR  GGUGAGGGCUCUACU**GCACUUUA** |
| [hsa-miR-526b-3p](http://www.mirbase.org/cgi-bin/mirna_entry.pl?acc=hsa-miR-526b-3p) | G**AAAGUGC**UUCCUUUUAGAGGC | Position 1109-1116 of HOXD11 3'UTR  GGUGAGGGCUCUACU**GCACUUUA** |
| hsa-miR-30a-5p | U**GUAAACA**UCCUCGACUGGAAG | Position 1138-1145 of HOXD11 3' UTR  AGGGCCAGGCCAGGC**UGUUUACA** |
| hsa-miR-489-3p | G**UGACAU**CACAUACACGGCAGC | Position 1480-1486 of HOXD11 3' UTR  CUUUGAACUUCCAAA**AUGUCAA**G |
| [hsa-miR-25-3p](http://www.mirbase.org/cgi-bin/mirna_entry.pl?acc=hsa-miR-25-3p) | C**AUUGCAC**UUGUCUCGGUCUGA | Position 1250-1257 of HOXD11 3' UTR  AGAGAUUUCCGCAAU**GUGCAAUA** |
| [hsa-miR-363-3p](http://www.mirbase.org/cgi-bin/mirna_entry.pl?acc=hsa-miR-363-3p) | A**AUUGCAC**GGUAUCCAUCUGUA | Position 1250-1257 of HOXD11 3' UTR  AGAGAUUUCCGCAAU**GUGCAAUA** |
| [hsa-miR-92a-3p](http://www.mirbase.org/cgi-bin/mirna_entry.pl?acc=hsa-miR-92a-3p) | U**AUUGCAC**UUGUCCCGGCCUGU | Position 1250-1257 of HOXD11 3' UTR  AGAGAUUUCCGCAAU**GUGCAAUA** |
| [hsa-miR-367-3p](http://www.mirbase.org/cgi-bin/mirna_entry.pl?acc=hsa-miR-367-3p) | A**AUUGCAC**UUUAGCAAUGGUGA | Position 1250-1257 of HOXD11 3' UTR  AGAGAUUUCCGCAAU**GUGCAAUA** |
| [hsa-miR-92b-3p](http://www.mirbase.org/cgi-bin/mirna_entry.pl?acc=hsa-miR-92b-3p) | U**AUUGCAC**UCGUCCCGGCCUCC | Position 1250-1257 of HOXD11 3' UTR  AGAGAUUUCCGCAAU**GUGCAAUA** |
| [hsa-miR-32-5p](http://www.mirbase.org/cgi-bin/mirna_entry.pl?acc=hsa-miR-32-5p) | U**AUUGCAC**AUUACUAAGUUGCA | Position 1250-1257 of HOXD11 3' UTR  AGAGAUUUCCGCAAUGUGCAAUA |
| [hsa-miR-142-5p](http://www.mirbase.org/cgi-bin/mirna_entry.pl?acc=hsa-miR-142-5p) | C**AUAAAGU**AGAAAGCACUACU | Position 1111-1117 of HOXD11 3' UTR  UGAGGGCUCUACUGC**ACUUUAU**G |
| [hsa-miR-5590-3p](http://www.mirbase.org/cgi-bin/mirna_entry.pl?acc=hsa-miR-5590-3p) | A**AUAAAGU**UCAUGUAUGGCAA | Position 1111-1117 of HOXD11 3' UTR  UGAGGGCUCUACUGC**ACUUUAU**G |
| hsa-miR-506-3p | G**UAAGGC**ACCCUUCUGAGUAGA | Position 1521-1527 of HOXD11 3' UTR  UCUGAAUAAUUAGGC**GCCUUAA**G |
| hsa-miR-216b-5p | A**AAUCUCU**GCAGGCAAAUGUGA | Position 1235-1241 of HOXD11 3' UTR  UCUGGUGGGCCCAAA**AGAGAUU**U |
| hsa-miR-137 | U**UAUUGC**UUAAGAAUACGCGUAG | Position 1252-1258 of HOXD11 3' UTR  AGAUUUCCGCAAUGU**GCAAUAA**A |

**Table S4.** Primers for qPCR amplification of target miRNA.

| **miRNA** | **qPCR amplification forward**  **primer (5' -> 3')** | **qPCR amplification reverse primer (5' -> 3')** |
| --- | --- | --- |
| hsa-miR-138-5p | GCGAGCTGGTGTTGTGAATC | AGTGCAGGGTCCGAGGTATT |
| hsa-miR-20b-5p | GCGCAAAGTGCTCATAGTGC | AGTGCAGGGTCCGAGGTATT |
| [hsa-miR-93-5p](http://www.mirbase.org/cgi-bin/mirna_entry.pl?acc=hsa-miR-93-5p) | CGCAAAGTGCTGTTCGTGC | AGTGCAGGGTCCGAGGTATT |
| [hsa-miR-519d-3p](http://www.mirbase.org/cgi-bin/mirna_entry.pl?acc=hsa-miR-519d-3p) | CGCAAAGTGCCTCCCTTT | AGTGCAGGGTCCGAGGTATT |
| [hsa-miR-17-5p](http://www.mirbase.org/cgi-bin/mirna_entry.pl?acc=hsa-miR-17-5p) | GCGCAAAGTGCTTACAGTGC | AGTGCAGGGTCCGAGGTATT |
| [hsa-miR-106a-5p](http://www.mirbase.org/cgi-bin/mirna_entry.pl?acc=hsa-miR-106a-5p) | CGCGAAAAGTGCTTACAGTGC | AGTGCAGGGTCCGAGGTATT |
| [hsa-miR-20a-5p](http://www.mirbase.org/cgi-bin/mirna_entry.pl?acc=hsa-miR-20a-5p) | GCGCGTAAAGTGCTTATAGTGC | AGTGCAGGGTCCGAGGTATT |
| [hsa-miR-106b-5p](http://www.mirbase.org/cgi-bin/mirna_entry.pl?acc=hsa-miR-106b-5p) | GCGCGTAAAGTGCTGACAGT | AGTGCAGGGTCCGAGGTATT |
| [hsa-miR-526b-3p](http://www.mirbase.org/cgi-bin/mirna_entry.pl?acc=hsa-miR-526b-3p) | CGCGGAAAGTGCTTCCTTTT | AGTGCAGGGTCCGAGGTATT |
| hsa-miR-30a-5p | CGCGTGTAAACATCCTCGAC | AGTGCAGGGTCCGAGGTATT |
| hsa-miR-489-3p | GCGCGGTGACATCACATACAC | AGTGCAGGGTCCGAGGTATT |
| [hsa-miR-25-3p](http://www.mirbase.org/cgi-bin/mirna_entry.pl?acc=hsa-miR-25-3p) | GCGCATTGCACTTGTCTCG | AGTGCAGGGTCCGAGGTATT |
| [hsa-miR-363-3p](http://www.mirbase.org/cgi-bin/mirna_entry.pl?acc=hsa-miR-363-3p) | GCGAATTGCACGGTATCCA | AGTGCAGGGTCCGAGGTATT |
| [hsa-miR-92a-3p](http://www.mirbase.org/cgi-bin/mirna_entry.pl?acc=hsa-miR-92a-3p) | GCGTATTGCACTTGTCCCG | AGTGCAGGGTCCGAGGTATT |
| [hsa-miR-367-3p](http://www.mirbase.org/cgi-bin/mirna_entry.pl?acc=hsa-miR-367-3p) | CGCGAATTGCACTTTAGCAA | AGTGCAGGGTCCGAGGTATT |
| [hsa-miR-92b-3p](http://www.mirbase.org/cgi-bin/mirna_entry.pl?acc=hsa-miR-92b-3p) | GCGTATTGCACTCGTCCCG | AGTGCAGGGTCCGAGGTATT |
| [hsa-miR-32-5p](http://www.mirbase.org/cgi-bin/mirna_entry.pl?acc=hsa-miR-32-5p) | CGCGCGTATTGCACATTACTAA | AGTGCAGGGTCCGAGGTATT |
| [hsa-miR-142-5p](http://www.mirbase.org/cgi-bin/mirna_entry.pl?acc=hsa-miR-142-5p) | GCGCGCATAAAGTAGAAAGC | AGTGCAGGGTCCGAGGTATT |
| [hsa-miR-5590-3p](http://www.mirbase.org/cgi-bin/mirna_entry.pl?acc=hsa-miR-5590-3p) | CGCGCGAATAAAGTTCATGTA | AGTGCAGGGTCCGAGGTATT |
| hsa-miR-506-3p | GCGGTAAGGCACCCTTCTG | AGTGCAGGGTCCGAGGTATT |
| hsa-miR-216b-5p | GCGAAATCTCTGCAGGCAA | AGTGCAGGGTCCGAGGTATT |
| hsa-miR-137 | CGCGCGTTATTGCTTAAGAATAC | AGTGCAGGGTCCGAGGTATT |

**Table S5.** Primer for Chromatin immunoprecipitation-qPCR.

| **Primer name** | **Forward sequence (5' -> 3')** | **Reverse sequence (5' -> 3')** |
| --- | --- | --- |
| FN1-P1 | ACTGTGGAAGTGGTGGACTCCT | CCTTTAAGATTCCCCCGCCTTG |
| FN1-P2 | ATGTGAGAAAGGGGCCGAAGG | GGACCAGCTGTGGGGAGAGAA |
| FN1-P3 | CTGAACTTCCCCGGGATCTGC | CACAGCTCCCTGTTCGGACTT |
| FN1-P4/P5 | AAAGAAAGGGAGCGGGATGGG | CGTCACCTCTCTTCGGGGTG |
| FN1-P6 | CACAAGTCCAGCCACTCCCTT | TGTGGGTTCGCAGCGAACAA |

**Table S6.** The differential-expressed target miRNAs in PSCC.

| **miRNA** | **Upregulated in:** | **Tumor log_2_**  **mean expression** | **Normal log_2_**  **mean expression** | ***p*-value** |
| --- | --- | --- | --- | --- |
| hsa-miR-138-5p | Normal | 1.03 | 3.88 | <0.001 |
| [hsa-miR-93-5p](http://www.mirbase.org/cgi-bin/mirna_entry.pl?acc=hsa-miR-93-5p) | Tumor | 9.50 | 8.45 | <0.01 |
| [hsa-miR-17-5p](http://www.mirbase.org/cgi-bin/mirna_entry.pl?acc=hsa-miR-17-5p) | Tumor | 7.45 | 4.69 | <0.001 |
| [hsa-miR-20a-5p](http://www.mirbase.org/cgi-bin/mirna_entry.pl?acc=hsa-miR-20a-5p) | Tumor | 4.72 | 3.99 | <0.01 |
| hsa-miR-30a-5p | Normal | 8.92 | 11.90 | <0.001 |
| hsa-miR-489-3p | Normal | 0.77 | 2.48 | <0.001 |
| [hsa-miR-32-5p](http://www.mirbase.org/cgi-bin/mirna_entry.pl?acc=hsa-miR-32-5p) | Normal | 0.68 | 2.15 | <0.001 |
| hsa-miR-506-3p | Normal | 0.09 | 0.98 | <0.01 |
| hsa-miR-137 | Tumor | 0.79 | 0.04 | <0.001 |

**Table S7.** The survival analysis of target miRNAs in PSCC.

| **miRNA** | **Clinical outcomes** | **Log-rank *p*-value** | **Hazard Ratio (95%Cl)** |
| --- | --- | --- | --- |
| hsa-miR-138-5p | better | 0.016 | 0.107 (0.031 - 0.370) |
| [hsa-miR-93-5p](http://www.mirbase.org/cgi-bin/mirna_entry.pl?acc=hsa-miR-93-5p) | poorer | 0.002 | 8.096 (2.225 - 29.46) |
| [hsa-miR-106a-5p](http://www.mirbase.org/cgi-bin/mirna_entry.pl?acc=hsa-miR-106a-5p) | better | 0.039 | 0.239 (0.069 - 0.827) |
| hsa-miR-30a-5p | poorer | 0.021 | 5.491 (1.565 - 19.27) |
| [hsa-miR-92a-3p](http://www.mirbase.org/cgi-bin/mirna_entry.pl?acc=hsa-miR-92a-3p) | better | 0.008 | 0.108 (0.031 - 0.373) |
| [hsa-miR-142-5p](http://www.mirbase.org/cgi-bin/mirna_entry.pl?acc=hsa-miR-142-5p) | poorer | 0.014 | 5.368 (1.533 - 18.79) |
| hsa-miR-137 | poorer | 0.042 | 3.282 (0.933 - 11.54) |

**Table S8.** The expression profile of hsa-miR-138-5p in different cancer types from the OncomiR database.

| **Cancer types** | **p-value** | **FDR** | **Upregulated in:** | **Tumor log^2^ mean expression** | **Normal log^2^ mean expression** |
| --- | --- | --- | --- | --- | --- |
| BLCA | 9.07e-03 | 2.42e-02 | Tumor | 1.21 | 0.21 |
| KICH | 2.60e-05 | 1.06e-04 | Normal | 0.29 | 2.09 |
| KIRC | 1.11e-19 | 1.53e-18 | Normal | 0.36 | 2.45 |
| KIRP | 1.18e-02 | 2.50e-02 | Normal | 1.92 | 2.73 |
| LUAD | 5.02e-05 | 3.05e-04 | Normal | 1.23 | 2.41 |
| LUSC | 1.03e-05 | 4.06e-05 | Normal | 1.45 | 2.82 |
| THCA | 2.27e-14 | 5.74e-13 | Normal | 5.13 | 6.89 |
| UCEC | 1.50e-02 | 3.18e-02 | Tumor | 0.90 | 0.00 |

BLCA: bladder urothelial carcinoma; KICH: kidney chromophobe; KIRC: kidney renal clear cell carcinoma; KIRP: kidney renal papillary cell carcinoma; LUAD: lung adenocarcinoma; LUSC: lung squamous cell carcinoma; THCA: thyroid carcinoma; UCEC: uterine corpus endometrial carcinoma
